# Evaluating genetic diversity and identifying priority conservation for seven Tibetan pig populations in China based on the mtDNA D-loop

**DOI:** 10.5713/ajas.19.0752

**Published:** 2020-01-13

**Authors:** Qianyun Ge, Caixia Gao, Yuan Cai, Ting Jiao, Jinqiang Quan, Yongbo Guo, Wangshan Zheng, Shengguo Zhao

**Affiliations:** 1 College of Animal Science & Technology, Gansu Agricultural University, Lanzhou 730070, China; 2 State Key Laboratory of Veterinary Biotechnology, Harbin Veterinary Research Institute, Chinese Academy of Agricultural Sciences, Harbin 150069, China; 3 College of Grassland, Gansu Agricultural University, Lanzhou 730070, China; 4 State Key Laboratory of Genetic Resources and Evolution, Kunming Institute of Zoology, Chinese Academy of Sciences, Kunming 650223, China

**Keywords:** Tibetan Pigs, mtDNA D-loop, Genetic Diversity, Conservation

## Abstract

**Objective:**

Tibetan pigs, an excellent species unique to China, face serious threats, which in turn affects the development and utilization of the outstanding advantages of plateau hypoxia adaptability and reduces their genetic diversity. Therefore, a discussion of measures to conserve this genetic resource is necessary. The method, based on genetic diversity, genetic divergence and total genetic contribution rate of population, reflects the priority conservation order and varies depending on the three different purposes of conservation.

**Methods:**

We analyzed mitochondrial DNA control region (D-loop) variation in 1,201 individuals from nine Tibetan pig populations across five provinces and downloaded 564 mtDNA D-loop sequences from three indigenous pig breeds in Qinghai, Sichuan, and Yunnan Provinces distributed near the Tibetan pigs.

**Results:**

We analyzed three different aspects: Changdu Tibetan pigs have the highest genetic diversity, and from the perspective of genetic diversity, the priority conservation is Changdu Tibetan pigs. Hezuo Tibetan pigs have the highest genetic contribution, so the priority conservation is Hezuo Tibetan pigs in the genetic contribution aspect. Rkaze Tibetan pigs were severely affected by indigenous pig breeds, so if considering from the perspective of introgression, the priority conservation is Rkaze Tibetan pigs.

**Conclusion:**

This study evaluated genetic diversity and comprehensively assessed conservation priority from three different aspects in nine Tibetan pig populations.

## INTRODUCTION

Tibetan pigs, an excellent species unique to China, are mainly distributed in the Tibetan Plateau area, which is the largest continuous high altitude ecosystem in the world, with an average altitude of more than 4,000 m [1]. These animals have a high status of genetic diversity in domestic pigs, but they also face serious threats, including loss habitat, because of increasing human population and activity, genetic introgression by crossbreeding with exotic breeds and intensifying production systems, which in turn affect the development and utilization of the outstanding advantages of plateau hypoxia adaptability and reduced genetic diversity. Although heterosis by crossing may improve economically important traits, undue crossbreeding may introduce DNA from other breeds and endanger the purity of some traditional local breeds [2], so genetic characterization of Tibetan pigs for conservation and rational use is therefore necessary and urgent. Due to differences in the size of populations, scarcity of funds for species conservation and conflict between conservation and economic development, deciding what and where to first conserve is an essential step in managing important species. Many researchers have studied issues, including genetic diversities in different populations [3], domestication centers [4], and migration history [5,6], that have helped us understand the local and worldwide domestication history by using mitochondrial DNA (mtDNA). Vertebrate mtDNA is capable of self-replication and is strictly maternally inherited without recombination during generational transmission. Although mtDNA contains many functional genes and the genome length and structure are very conservative, but the primary structure changes are very active. mtDNA contains the displacement (D)-loop, which contains regulatory sequences controlling both replication and transcription of mtDNA, is the fastest-evolving region in mtDNA. Due to the high variation of mtDNA sequences, it can be effectively used to establish evolutionary relationships within species and between different populations. In this study, therefore, the genetic diversity of Tibetan pigs in nine populations across five provinces was comprehensively analyzed and assessed using the mtDNA D-loop, and priorities for conservation were discussed. The results of the study will contribute to the conservation and sustainable use of resources.

## MATERIALS AND METHODS

### Sampling and sequencing

Blood samples were collected from a total of 728 Tibetan pigs in seven different populations of Tibetan pigs where the samples had not been adequately collected before: Ganzi (79), Diqing (83), Linzhi (122), Shannan (72), Changdu (73), Hezuo (193), and Qinghai (106). All animal work was conducted according to the Institutional Animal Care and Use Committee (IACUC) and was approved by the Animal Care Committee of Gansu Agricultural University. Considering the limited quantity of testing sequences, an additional dataset of 473 samples was used to complement the data on representative Tibetan pigs to obtain high coverage of Tibetan pigs. A total of 564 mtDNA D-loop sequences from three indigenous pig breeds in Qinghai, Sichuan and Yunnan Provinces, which are located near where Tibetan pigs are distributed, were downloaded from GenBank and used as a reference set in this study. All sample summaries are listed in Table 1 (a list of collected and supplementary animals, with GenBank accession number and other detailed information, is provided in an editable format in Supplementary Table S1).

The D-loop region was amplified directly from the genomic DNA by polymerase chain reaction (PCR) using the primers 5′-CCAAAAACAAAGCAGAGTGTAC-3′ and 5′-CGTTA TGAGCTACCGTTATA-3′. PCR was carried out in 25 μL volumes and contained 12.5 μL of 2× Eco Taq PCR Supermix containing 1 U Taq polymerase, 500 mμ dNTPs, and 10× Taq buffer (Beijing TransGen Biotech Co., Ltd., Beijing, China), 0.1 μg of template DNA, 0.4 μL of each primer at 10 pmol/mL and 11.6 μL of ddH_2_O. The cycling conditions were initial denaturation at 94°C for 5 min, followed by 33 cycles of 94°C for 30 s, 56°C for 30 s and 72°C for 30 s and a final extension for 5 min at 72°C [7]. Amplified DNA fragments were purified following agarose gel electrophoresis and sequenced using the ABI 3130 DNA sequencer (Applied Biosystems, Foster City, CA, USA) [7].

### Data analysis

Original sequence data were obtained using the ABI PRISM DNA sequencer software. Sequences were edited and aligned using ClustalX 1.81 [8]. MEGA 7.0 was used to collect sequences [9]. DnaSP 5.0 software was used to analyze the haplotypes and genetic diversity [7]. Correlation analysis and principal component analysis (PCA) were investigated by SPSS 19.0.

## RESULTS

### Genetic diversity analysis

**Figure 1:**
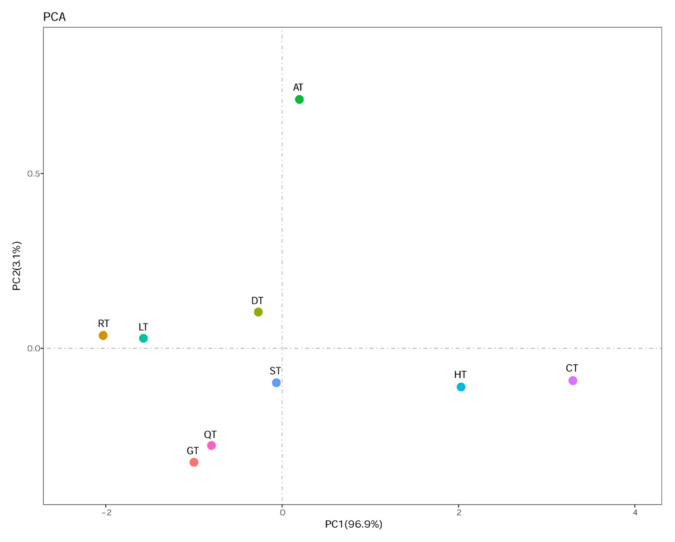
Principal component analysis (PCA). The 431-bp D-loop region of mtDNA was used to analyze the genetic diversity for all 1,201 sequences in Tibetan pigs (Diqing, n = 178; Linzhi, n = 241; Shannan, n = 91; Changdu, n = 90; Rkaze, n = 24; Aba, n = 70; Ganzi, n = 133; Hezuo, n = 268; Qinghai, n = 106). The cumulative contribution rate is in the parentheses.

The 431-bp D-loop region of mtDNA was obtained. Haplotype diversity (Hd), nucleotide diversity (Pi) and average number of nucleotide differences (K) are the basic parameters used to assess genetic diversity. PCA is a statistical procedure used to reduce the dimensionality of a dataset by transformation to a new set of variables (the principal components) to summarize the features of the data [10]. Genetic diversity was analyzed by PCA, and the results are shown in Figure 1. We extracted two principal components (PCA1 and PCA2) defined by principal component factor scores based on a components matrix from Hd, Pi, and K. The cumulative contribution rate of PCA1 was 96.9%, which indicates that it can reflect 96.9% of the original variables and can meet the application requirements. At this point, we made a decision to use PCA1 to reflect genetic diversity. We obtained the result from Figure 1, in that the highest genetic diversity was in Changdu Tibetan pigs (CT) followed by Hezuo Tibetan pigs (HT) and Aba Tibetan pigs (AT), while Rkaze Tibetan pigs (RT) have the lowest genetic diversity.

**Figure 2:**
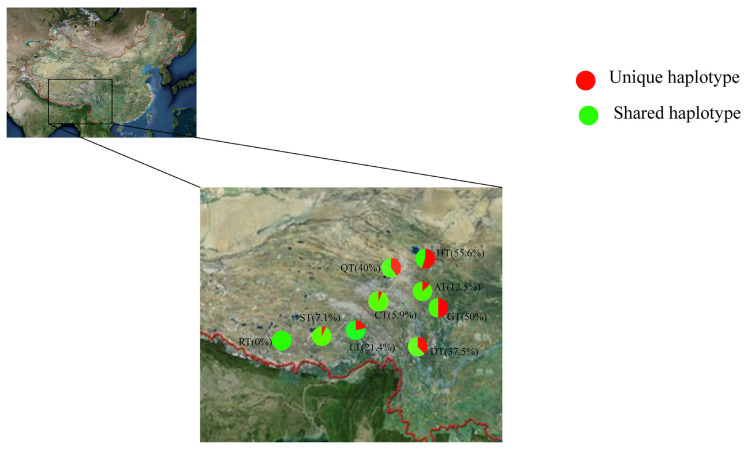
Comparison of the unique haplotype frequency of Tibetan pigs. Each pie chart shows the ratio of unique haplotypes and shared haplotypes among nine Tibetan pig populations.

A total of 38 unique haplotypes were identified in nine Tibetan pig populations. The HT have the largest number of unique haplotypes (15) and the highest ratio of unique haplotypes (55.6%). RT have no unique haplotypes (Figure 2). Thus, the HT population is the most distinctive, followed by the Ganzi Tibetan (GT) and Qinghai Tibetan pig (QT) populations.

### Genetic contribution analysis

To synthesize genetic distinctiveness and diversity, Petit et al [11] proposed the approach of genetic contribution. The contributions of genetic diversity (R_S(k)_) and genetic distinctiveness (R_D(K)_) are combined to obtain the total genetic contribution (R_T(K)_) of the kth population.

$$\begin{array}{l} {\text{R}}_{\text{S(k)}}=\frac{R_{k}}{n}\\ {\text{R}}_{\text{D(k)}}=\sum_{i}^{R_{k}}\frac{n-n_{i}}{{\text{nn}}_{i}}\\ {\text{R}}_{\text{T(k)}}={\text{R}}_{\text{S}(\text{k})}+{\text{R}}_{\text{D}(\text{k})}=\sum_{i}^{R_{k}}\frac{1}{n_{i}} \end{array}$$

where n represents the total number of populations studied, and n_i_ represents the number of populations with the ith haplotype. Similarly, the rates of contribution attributed to genetic variation (C_S(K)_) and genetic distinctiveness (C_D(k)_) to the total genetic contribution rate (C_T(k)_) of the Kth population with R_K_ haplotypes are obtained using the following formulas:

$$\begin{array}{l} {\text{C}}_{\text{RS(k)}}=\frac{R_{S(k)}-{\overset{ˋ}{R}}_{S}}{R_{T}}\\ {\text{C}}_{\text{RD(k)}}=\frac{R_{D(k)}-{\bar{R}}_{D}}{R_{T}}\\ {\text{C}}_{\text{RT(k)}}=\frac{R_{T(k)}-{\overset{ˋ}{R}}_{T}}{R_{T}} \end{array}$$

where R_T_ represents the total number of haplotypes, *R̄_S_* = ∑*R_S_*_(_*_k_*_)_/*n*, *R̄_D_* = ∑*R_D_*_(_*_k_*_)_/*n*, *R̄_D_* = ∑*R_T_*_(_*_k_*_)_/*n*. The total contribution rate C_RT(k)_ can be partitioned into two components, C_RS(k)_, which is the rate of contribution of the kth population due to its own diversity, and C_RD(k)_, the contribution due to its divergence, i.e., C_RT(k)_ = C_RS(k)_+C_RD(k)_, ∑*C_RS_*_(_*_k_*_)_ = 0, ∑*C_RD_*_(_*_k_*_)_ = 0, ∑*C_RT_*_(_*_k_*_)_ = 0.

The values obtained for R_S(k)_ and R_D(K)_ (and therefore R_T(K)_) were highest for the HT and lowest for the RT (Table 2). The values of C_S(K)_, C_D(K)_, and C_T(k)_ provide relative overall criteria for setting priorities for conservation of Tibetan pig populations. The highest values of C_S(K)_, C_D(K)_, and C_T(K)_ were in HT (Figure 2) and lowest in RT.

### Shared haplotypes between Tibetan pigs and indigenous breeds

**Figure 3:**
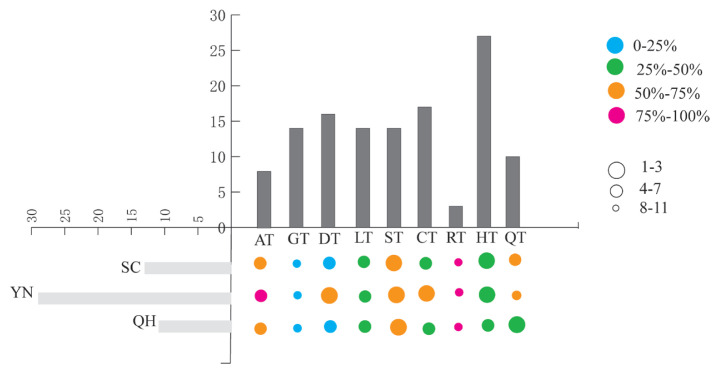
Shared haplotypes with indigenous pigs in Tibetan pigs. Each pie chart represents the shared haplotypes, and different sizes represent the number of shared haplotypes. Different colors represent the proportion of shared haplotypes in the total haplotypes of each Tibetan pig population.

Haplotypes in 1201 individuals from nine Tibetan pig populations and 564 individuals from three indigenous breeds were identified (Table 1). Sixty haplotypes were identified in Tibetan pigs, and 40 haplotypes were identified in indigenous pigs. Twenty-three shared haplotypes were identified between indigenous and Tibetan pigs distributed among 511 indigenous and 1,088 Tibetan pigs. The shared haplotypes with number and degree of introgression to Tibetan pig haplotypes are shown in Figure 3. We can see that the GT haplotypes were minimally introgressed and the Diqing Tibetan (DT) haplotypes were minimally introgressed by the Sichuan indigenous pig and Qinghai indigenous pig haplotypes. The RT haplotypeswere greatly introgressed, and the AT haplotypes were greatly introgressed by the haplotypes of Yunnan indigenous pig. The percentage of the number of Tibetan pigs with shared haplotypes and the total of Tibetan pigs in each population (Sc/S) showed the degree of Tibetan pigs affected by indigenous pigs (Table 3). The average percentage of Sc/S was 92.7% and ranged from 84.7% to 100% (Table 3). Our data showed that Tibetan pigs were greatly impacted by indigenous breeds (from the Qinghai, Sichuan and Yunnan Provinces of China).

## DISCUSSION

### Genetic diversity of the Tibetan pig population

The genetic diversity of global livestock populations is declining [12]. Our study evaluated the genetic diversity in Tibetan pig populations. We analyzed the mtDNA D-loop for haplotypes of 1,201 Tibetan pig samples in nine populations (Table 1), and 60 haplotypes were identified, including 38 unique haplotypes. We also analyzed genetic diversity by PCA with a synthesized assessment score (Fz) score based on basic parameters (Hd, Pi, and K). Hd reflects haplotype uniqueness in a population [13]. Pi measures the degree of polymorphism within a population [14]. Pi and K measure the degree of intrapopulation haplotype mutation [15]. The analysis showed that the genetic diversity in CT was highest and lowest in RT in nine Tibetan pig populations, which was consistent with the analysis of the parameters Hd, Pi, and K. Fz was in the high range and higher on average, indicating that Tibetan pigs have a high status of genetic diversity in domestic pigs. In conclusion, the genetic diversity observed in Tibetan pigs highlights that it is an important genetic resource that is important for continued reproduction. Porcine genetic diversity could also be useful for sourcing future breeds for livestock production and supplementing biodiversity databasesaccumulated from populations and breeds around the world [16].

Genetic distinctiveness is an important factor that is used when populations are selected for conservation. The highest priority for conservation in the population often has the highest genetic distinctiveness [17]. A previous study selected distinct populations of Spring Monkey for priority conservation using evolutionarily significant units, which is a measure of genetic distinctiveness [18]. In this study, a total of 38 unique haplotypes were detected in nine Tibetan pig populations. The HT have the largest number of unique haplotypes (15) and the highest ratio of unique haplotypes (55.6%). The RT have no unique haplotypes. The results suggest that the HT population has the highest priority for conservation in distinctive aspects, followed by the GT and QT populations.

### Genetic contribution

Genetic diversity and genetic distinctiveness highlight two different aspects of genetic diversity; these two aspects are both important for the conservation of species. It is easy to misjudge using only one aspect, which is not conducive to the effective conservation of species. Therefore, considering the role of both aspects is necessary. Based on this, Petit et al [11] put forth the approach of genetic contribution, which considers genetic diversity and distinctiveness. This approach appears to be the most appropriate for selecting populations for conservation [19,20]. The contributions of genetic diversity (R_S(K)_) and genetic distinctiveness (R_D(K)_) are combined to obtain the total genetic contribution (R_T(K)_) of the kth population. C_RS(K)_ and C_RD(k)_ reflect the effects of population k in maintaining genetic diversity within the population and genetic divergence between populations, while C_RT(k)_ reflects the combined effect of the two, which is the effect of maintaining the overall allelic richness of the species. The calculation of these three values reflects the measure of the relative contribution rate of the genetic diversity, distinctiveness and total genetic contribution by comparing the degree of difference between the genetic contribution rate of each population and the average genetic contribution among nine populations, based on genetic diversity, genetic divergence and total genetic contribution rate of population.

The order of priority conservation of a population based on genetic diversity and genetic distinctive were discussed respectively earlier in this study and did not take into account the combined effect of both aspects. Therefore, to fully consider the two factors of genetic diversity and genetic distinctiveness, the order of priority conservation of population was evaluated based on the model of genetic contribution rate.

**Figure 4:**
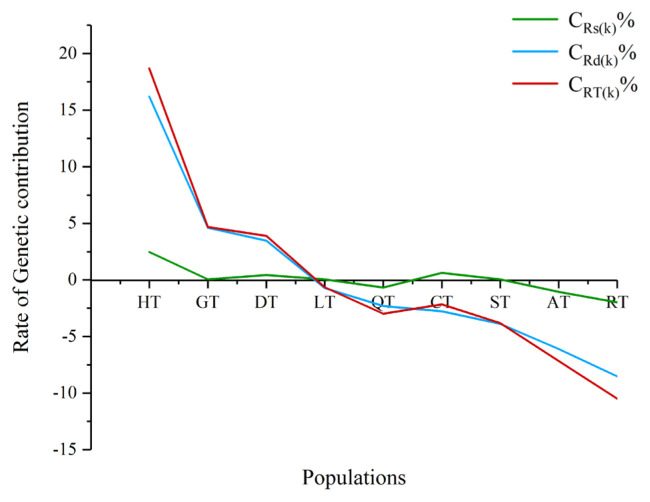
Rate of genetic contribution in nine Tibetan pig populations.

As shown in Figure 4, the C_RD(k)_ is generally consistent with the C_RT(k)_ curve, while the C_RS(k)_ is similar to the previous two curves, but the values are quite different. This indicates that the genetic distinctiveness has a greater contribution rate to the overall genetic effect, while the contribution rate of the genetic diversity effect is smaller; that is, the genetic distinctiveness has the greatest influence and the most sensitive effect on the overall genetic effect. Ranking of nine populations prioritized based on overall genetic effects, the overall genetic effect of HT, GT, and DT was positive, indicating that the genetic contribution of Tibetan pigs in these populations is higher than the average genetic contribution, and its presence increases the overall allele richness of Tibetan pigs and is worthy of conservation. The values in HT were higher than those of other populations, indicating that the HT population could contribute the most to improving the genetic variation and haplotype richness of Tibetan pigs, followed by the GT and DT populations.

In summary, based on the C_RS(k)_, C_RD(k)_, or C_RT(k)_ values, the pre-existing population conservation value is large; in particular, those populations with positive C_RS(k)_, C_RS(k)_, or C_RT(k)_ should be given priority protection.

### The introgression of Tibetan pigs

Shared haplotypes were identified in 92.7% of Tibetan pigs on average, indicating that Tibetan pigs are severely introgressed by the indigenous pig breeds, which are distributed in the surrounding areas. In total, 38.3% (23/60) of haplotypes that were shared with indigenous pigs were identified in 90.6% (1,088/1,201) of Tibetan pigs. Shared haplotypes between indigenous and Tibetan pigs showed an unequal global distribution (Table 3). The frequency of shared haplotypes was lowest in HT, while it was highest in RT, where the Tibetan pig population was relatively greatly affected by indigenous pigs. To explore the conservation of Tibetan pig genetic resources from the perspective of introgression from indigenous breeds to Tibetan pigs, we should first consider the populations that are seriously affected by indigenous breeds. All individuals in the RT population analysis in this study shared haplotypes with indigenous breeds, indicating that the RT was more affected by indigenous influences.

## CONCLUSION

Priority conservation order varies depending on the purpose of conservation. According to the principle of genetic diversity, the first priority in conservation is CT. According to the principle of genetic contribution rate, the first priority in conservation is HT. Tibetan pigs of different populations are threatened by indigenous pig breeds to varying degrees, RT is more affected by indigenous breeds, and the first priority in conservation is RT in introgression.

## Figures and Tables

**Table 1:** Characteristics of samples

Breed/Population	Abbreviation	Category	Sample size	Sampling site
Aba Tibetan pig	AT	Tibetan	70	Aba, Sichuan
Ganzi Tibetan pig	GT	Tibetan	133	Ganzi, Sichuan
Diqing Tibetan pig	DT	Tibetan	178	Diqing, Yunan
Linzhi Tibetan pig	LT	Tibetan	241	Linzhi, Tibet
Shannan Tibetan pig	ST	Tibetan	91	Shannan, Tibet
Changdu Tibetan pig	CT	Tibetan	90	Changdu, Tibet
Rkaze Tibetan pig	RT	Tibetan	24	Rkaze, Tibet
Hezuo Tibetan pig	HT	Tibetan	268	Hezuo, Gansu
Qinghai Tibetan pig	QT	Tibetan	106	Qinghai
Qinghai indigenous pig	QH	Indigenous	115	Qinghai
Yunnan indigenous pig	YN	Indigenous	219	Yunnan
Sichuan indigenous pig	SC	Indigenous	230	Sichuan

**Table 2:** Genetic contributions of nine Tibetan pig populations

Items	h	R_S(k)_	R_D(k)_	R_T(k)_
Aba Tibetan pig	8	0.8889	1.4857143	2.3746032
Ganzi Tibetan pig	14	1.5556	7.9166667	9.4722222
Diqing Tibetan pig	16	1.7778	7.2301587	9.0079365
Linzhi Tibetan pig	14	1.5556	4.7261905	6.281746
Shannan Tibetan pig	14	1.5556	2.8190476	4.3746032
Changdu Tibetan pig	17	1.8889	3.4857143	5.3746032
Rkaze Tibetan pig	3	0.3333	0.031746	0.3650794
Hezuo Tibetan pig	27	3.0000	14.874603	17.874603
Qinghai Tibetan pig	10	1.1111	3.7634921	4.8746032

R_S(k)_, genetic diversity; R_D(k)_, genetic distinctiveness; R_T(k)_, total genetic contribution.

**Table 3:** Analysis of Tibetan pig haplotypes shared with indigenous pigs

Population	Sc	S	Sc/S (%)
Aba	68	70	97.14
Ganzi	112	133	84.21
Diqing	158	178	88.76
Linzhi	225	241	93.36
Shannan	87	91	95.60
Changdu	84	90	93.33
Rikeze	24	24	100.00
Hezuo	227	268	84.70
Qinghai	103	106	97.17

Sc, number of Tibetan pigs sharing haplotypes with indigenous pigs; S, number of Tibetan pigs.
