# Supplementary material for: Evaluating genetic diversity and identifying priority conservation for seven Tibetan pig populations in China based on the mtDNA D-loop
Source: Asian-Australas J Anim Sci. 2020 Jan 13;33(12):1905–11. doi: 10.5713/ajas.19.0752 (PMC7649404; doi:10.5713/ajas.19.0752)
Supplement: Supplementary file 1 [file ajas-19-0752-suppl.pdf]

Table S1. Information on sampling (Indigenous pigs).

Sample IC	Accession	Breed/status	Location	References
QH001	HM026670	Bamei	Qinghai	Byeong-Woo Kim et al.2011
QH002	DQ379180.2	Huzhu	Qinghai	Fang,M et al. 2006
QH003	DQ379179.2	Huzhu	Qinghai	Fang,M et al. 2006
QH004	DQ379164.2	Bamei	Qinghai	Fang,M et al. 2006
QH005	DQ379163.2	Bamei	Qinghai	Fang,M et al. 2006
QH006	DQ379104.2	Bamei	Qinghai	Fang,M et al. 2006
QH007	DQ379103.2	Bamei	Qinghai	Fang,M et al. 2006
QH008	DQ152888.2	Huzhu	Qinghai	Fang,M et al. 2006
QH009	DQ152887.2	Huzhu	Qinghai	Fang,M et al. 2006
QH010	DQ152886.2	Huzhu	Qinghai	Fang,M et al. 2006
QH011	DQ152879.2	Bamei	Qinghai	Fang,M et al. 2006
QH012	EF545588	Huzu	Qinghai	Gui-Sheng Wu et al.2007
QH013	EF545587	Huzu	Qinghai	Gui-Sheng Wu et al.2007
QH014	EF545583	Bamei	Qinghai	Gui-Sheng Wu et al.2007
QH015	DQ496466	Huzu	Qinghai	Gui-Sheng Wu et al.2007
QH016	DQ496465	Huzu	Qinghai	Gui-Sheng Wu et al.2007
QH017	DQ496464	Huzu	Qinghai	Gui-Sheng Wu et al.2007
QH018	DQ496463	Huzu	Qinghai	Gui-Sheng Wu et al.2007
QH019	DQ496462	Huzu	Qinghai	Gui-Sheng Wu et al.2007
QH020	DQ496460	Huzu	Qinghai	Gui-Sheng Wu et al.2007
QH021	DQ496459	Huzu	Qinghai	Gui-Sheng Wu et al.2007
QH022	DQ496458	Huzu	Qinghai	Gui-Sheng Wu et al.2007
QH023	DQ496457	Huzu	Qinghai	Gui-Sheng Wu et al.2007
QH024	DQ496456	Huzu	Qinghai	Gui-Sheng Wu et al.2007
QH025	DQ496455	Huzu	Qinghai	Gui-Sheng Wu et al.2007
QH026	DQ496454	Huzu	Qinghai	Gui-Sheng Wu et al.2007
QH027	DQ496453	Huzu	Qinghai	Gui-Sheng Wu et al.2007
QH028	DQ496452	Huzu	Qinghai	Gui-Sheng Wu et al.2007
QH029	DQ496451	Huzu	Qinghai	Gui-Sheng Wu et al.2007
QH030	DQ496292	Bamei	Qinghai	Gui-Sheng Wu et al.2007
QH031	DQ496291	Bamei	Qinghai	Gui-Sheng Wu et al.2007
QH032	DQ496290	Bamei	Qinghai	Gui-Sheng Wu et al.2007
QH033	DQ496289	Bamei	Qinghai	Gui-Sheng Wu et al.2007
QH034	DQ496288	Bamei	Qinghai	Gui-Sheng Wu et al.2007
QH035	DQ496287	Bamei	Qinghai	Gui-Sheng Wu et al.2007
QH036	DQ496286	Bamei	Qinghai	Gui-Sheng Wu et al.2007
QH037	DQ496285	Bamei	Qinghai	Gui-Sheng Wu et al.2007
QH038	DQ496284	Bamei	Qinghai	Gui-Sheng Wu et al.2007
QH039	DQ496283	Bamei	Qinghai	Gui-Sheng Wu et al.2007
QH040	DQ496282	Bamei	Qinghai	Gui-Sheng Wu et al.2007
QH041	DQ496265	Huzu	Qinghai	Gui-Sheng Wu et al.2007
QH042	DQ496264	Huzu	Qinghai	Gui-Sheng Wu et al.2007
QH043		Bamei	Qinghai	Tiantuan Jiang
QH044		Bamei	Qinghai	Tiantuan Jiang
QH045		Bamei	Qinghai	Tiantuan Jiang
QH046		Bamei	Qinghai	Tiantuan Jiang
QH047		Bamei	Qinghai	Tiantuan Jiang
QH048		Bamei	Qinghai	Tiantuan Jiang
QH049		Bamei	Qinghai	Tiantuan Jiang
QH050		Bamei	Qinghai	Tiantuan Jiang
QH051		Bamei	Qinghai	Tiantuan Jiang
QH052		Bamei	Qinghai	Tiantuan Jiang
QH053		Bamei	Qinghai	Tiantuan Jiang
QH054		Bamei	Qinghai	Tiantuan Jiang
QH055		Bamei	Qinghai	Tiantuan Jiang

QH056	Bamei	Qinghai	Tiantuan Jiang
QH057	Bamei	Qinghai	Tiantuan Jiang
QH058	Bamei	Qinghai	Tiantuan Jiang
QH059	Bamei	Qinghai	Tiantuan Jiang
QH060	Bamei	Qinghai	Tiantuan Jiang
QH061	Bamei	Qinghai	Tiantuan Jiang
QH062	Bamei	Qinghai	Tiantuan Jiang
QH063	Bamei	Qinghai	Tiantuan Jiang
QH064	Bamei	Qinghai	Tiantuan Jiang
QH065	Bamei	Qinghai	Tiantuan Jiang
QH066	Bamei	Qinghai	Tiantuan Jiang
QH067	Bamei	Qinghai	Tiantuan Jiang
QH068	Bamei	Qinghai	Tiantuan Jiang
QH069	Bamei	Qinghai	Tiantuan Jiang
QH070	Bamei	Qinghai	Tiantuan Jiang
QH071	Bamei	Qinghai	Tiantuan Jiang
QH072	Bamei	Qinghai	Tiantuan Jiang
QH073	Bamei	Qinghai	Tiantuan Jiang
QH074	Bamei	Qinghai	Tiantuan Jiang
QH075	Bamei	Qinghai	Tiantuan Jiang
QH076	Bamei	Qinghai	Tiantuan Jiang
QH077	Bamei	Qinghai	Tiantuan Jiang
QH078	Bamei	Qinghai	Tiantuan Jiang
QH079	Bamei	Qinghai	Tiantuan Jiang
QH080	Bamei	Qinghai	Tiantuan Jiang
QH081	Bamei	Qinghai	Tiantuan Jiang
QH082	Bamei	Qinghai	Tiantuan Jiang
QH083	Bamei	Qinghai	Tiantuan Jiang
QH084	Bamei	Qinghai	Tiantuan Jiang
QH085	Bamei	Qinghai	Tiantuan Jiang
QH086	Bamei	Qinghai	Tiantuan Jiang
QH087	Bamei	Qinghai	Tiantuan Jiang
QH088	Bamei	Qinghai	Tiantuan Jiang
QH089	Bamei	Qinghai	Tiantuan Jiang
QH090	Bamei	Qinghai	Tiantuan Jiang
QH091	Bamei	Qinghai	Tiantuan Jiang
QH092	Bamei	Qinghai	Tiantuan Jiang
QH093	Bamei	Qinghai	Tiantuan Jiang
QH094	Bamei	Qinghai	Tiantuan Jiang
QH095	Bamei	Qinghai	Tiantuan Jiang
QH096	Bamei	Qinghai	Tiantuan Jiang
QH097	Bamei	Qinghai	Tiantuan Jiang
QH098	Bamei	Qinghai	Tiantuan Jiang
QH099	Bamei	Qinghai	Tiantuan Jiang
QH100	Bamei	Qinghai	Tiantuan Jiang
QH101	Bamei	Qinghai	Tiantuan Jiang
QH102	Bamei	Qinghai	Tiantuan Jiang
QH103	Bamei	Qinghai	Tiantuan Jiang
QH104	Bamei	Qinghai	Tiantuan Jiang
QH105	Bamei	Qinghai	Tiantuan Jiang
QH106	Bamei	Qinghai	Tiantuan Jiang
QH107	Bamei	Qinghai	Tiantuan Jiang
QH108	Bamei	Qinghai	Tiantuan Jiang
QH109	Bamei	Qinghai	Tiantuan Jiang
QH110	Bamei	Qinghai	Tiantuan Jiang

QH111		Bamei	Qinghai	Tiantuan Jiang
QH112		Bamei	Qinghai	Tiantuan Jiang
QH113		Bamei	Qinghai	Tiantuan Jiang
QH114		Bamei	Qinghai	Tiantuan Jiang
QH115		Bamei	Qinghai	Tiantuan Jiang
YNW001	DQ496327	Baoshan	Yunnan, Ba	Gui-Sheng Wu et al.2007
YNW002	DQ496326	Baoshan	Yunnan, Ba	Gui-Sheng Wu et al.2007
YNW003	DQ496325	Baoshan	Yunnan, Ba	Gui-Sheng Wu et al.2007
YNW004	DQ496324	Baoshan	Yunnan, Ba	Gui-Sheng Wu et al.2007
YNW005	DQ496323	Baoshan	Yunnan, Ba	Gui-Sheng Wu et al.2007
YNW006	DQ496322	Baoshan	Yunnan, Ba	Gui-Sheng Wu et al.2007
YNW007	DQ496321	Baoshan	Yunnan, Ba	Gui-Sheng Wu et al.2007
YNW008	DQ496320	Baoshan	Yunnan, Ba	Gui-Sheng Wu et al.2007
YNW009	DQ496319	Baoshan	Yunnan, Ba	Gui-Sheng Wu et al.2007
YNW010	DQ496318	Baoshan	Yunnan, Ba	Gui-Sheng Wu et al.2007
YNW011	DQ496317	Baoshan	Yunnan, Ba	Gui-Sheng Wu et al.2007
YNW012	DQ496316	Baoshan	Yunnan, Ba	Gui-Sheng Wu et al.2007
YNW013	DQ496315	Baoshan	Yunnan, Ba	Gui-Sheng Wu et al.2007
YNW014	DQ496314	Baoshan	Yunnan, Ba	Gui-Sheng Wu et al.2007
YNW015	DQ496313	Baoshan	Yunnan, Ba	Gui-Sheng Wu et al.2007
YNW016	DQ496312	Baoshan	Yunnan, Ba	Gui-Sheng Wu et al.2007
YNW017	DQ496311	Baoshan	Yunnan, Ba	Gui-Sheng Wu et al.2007
YNW018	DQ496310	Baoshan	Yunnan, Ba	Gui-Sheng Wu et al.2007
YNW019	DQ496309	Baoshan	Yunnan, Ba	Gui-Sheng Wu et al.2007
YNW020	HQ148551	Baoshan	Yunnan, Ba	Gou, X et al. 2011
YNW021	HQ148550	Baoshan	Yunnan, Ba	Gou, X et al. 2011
YNW022	HQ148549	Baoshan	Yunnan, Ba	Gou, X et al. 2011
YNW023	HQ148548	Baoshan	Yunnan, Ba	Gou, X et al. 2011
YNW024	HQ148547	Baoshan	Yunnan, Ba	Gou, X et al. 2011
YNW025	HQ148546	Baoshan	Yunnan, Ba	Gou, X et al. 2011
YNW026	HQ148545	Baoshan	Yunnan, Ba	Gou, X et al. 2011
YNW027	HQ148544	Baoshan	Yunnan, Ba	Gou, X et al. 2011
YNW028	HQ148543	Baoshan	Yunnan, Ba	Gou, X et al. 2011
YNW029	HQ148542	Baoshan	Yunnan, Ba	Gou, X et al. 2011
YNW030	HQ148541	Baoshan	Yunnan, Ba	Gou, X et al. 2011
YNW031	HQ148540	Baoshan	Yunnan, Ba	Gou, X et al. 2011
YNW032	HQ148539	Baoshan	Yunnan, Ba	Gou, X et al. 2011
YNW033	HQ148538	Baoshan	Yunnan, Ba	Gou, X et al. 2011
YNW034	HQ148537	Baoshan	Yunnan, Ba	Gou, X et al. 2011
YNW035	HQ148536	Baoshan	Yunnan, Ba	Gou, X et al. 2011
YNW036	HQ148535	Baoshan	Yunnan, Ba	Gou, X et al. 2011
YNW037	HQ148534	Baoshan	Yunnan, Ba	Gou, X et al. 2011
YNW038	HQ148533	Baoshan	Yunnan, Ba	Gou, X et al. 2011
YNW039	DQ496580	Mingguang	Yunnan	Gui-Sheng Wu et al.2007
YNW040	DQ496579	Mingguang	Yunnan	Gui-Sheng Wu et al.2007
YNW041	DQ496578	Mingguang	Yunnan	Gui-Sheng Wu et al.2007
YNW042	DQ496577	Mingguang	Yunnan	Gui-Sheng Wu et al.2007
YNW043	DQ496576	Mingguang	Yunnan	Gui-Sheng Wu et al.2007
YNW044	DQ496575	Mingguang	Yunnan	Gui-Sheng Wu et al.2007
YNW045	DQ496574	Mingguang	Yunnan	Gui-Sheng Wu et al.2007
YNW046	DQ496573	Mingguang	Yunnan	Gui-Sheng Wu et al.2007
YNW047	DQ496572	Mingguang	Yunnan	Gui-Sheng Wu et al.2007
YNW048	DQ496553	Mingguang	Yunnan	Gui-Sheng Wu et al.2007
YNW049	DQ496552	Mingguang	Yunnan	Gui-Sheng Wu et al.2007
YNW050	DQ496551	Mingguang	Yunnan	Gui-Sheng Wu et al.2007
YNW051	DQ496550	Mingguang	Yunnan	Gui-Sheng Wu et al.2007
YNW052	DQ496549	Mingguang	Yunnan	Gui-Sheng Wu et al.2007
YNW053	DQ496548	Mingguang	Yunnan	Gui-Sheng Wu et al.2007

YNW054	DQ496423	Fugong	Yunnan	Gui-Sheng Wu et al.2007
YNW055	HQ148532	Gaoligongshan	Yunnan,Nu	Gou,X et al. 2011
YNW056	HQ148531	Gaoligongshan	Yunnan,Nu	Gou,X et al. 2011
YNW057	HQ148530	Gaoligongshan	Yunnan,Nu	Gou,X et al. 2011
YNW058	HQ148529	Gaoligongshan	Yunnan,Nu	Gou,X et al. 2011
YNW059	HQ148528	Gaoligongshan	Yunnan,Nu	Gou,X et al. 2011
YNW060	HQ148527	Gaoligongshan	Yunnan,Nu	Gou,X et al. 2011
YNW061	HQ148526	Gaoligongshan	Yunnan,Nu	Gou,X et al. 2011
YNW062	HQ148525	Gaoligongshan	Yunnan,Nu	Gou,X et al. 2011
YNW063	HQ148524	Gaoligongshan	Yunnan,Nu	Gou,X et al. 2011
YNW064	HQ148523	Gaoligongshan	Yunnan,Nu	Gou,X et al. 2011
YNS001	GQ220329	Dahe		Su,X.X
YNS002	DQ496363	Dahe	Yunnan	Gui-Sheng Wu et al.2007
YNS003	DQ496362	Dahe	Yunnan	Gui-Sheng Wu et al.2007
YNS004	DQ496361	Dahe	Yunnan	Gui-Sheng Wu et al.2007
YNS005	DQ496360	Dahe	Yunnan	Gui-Sheng Wu et al.2007
YNS006	DQ496359	Dahe	Yunnan	Gui-Sheng Wu et al.2007
YNS007	DQ496358	Dahe	Yunnan	Gui-Sheng Wu et al.2007
YNS008	DQ496461	Dahe	Yunnan	Gui-Sheng Wu et al.2007
YNS010	DQ496391	Diannanxiao’	Yunnan	Gui-Sheng Wu et al.2007
YNS011	DQ496390	Diannanxiao’	Yunnan	Gui-Sheng Wu et al.2007
YNS012	DQ496389	Diannanxiao’	Yunnan	Gui-Sheng Wu et al.2007
YNS013	DQ496388	Diannanxiao’	Yunnan	Gui-Sheng Wu et al.2007
YNS014	DQ496387	Diannanxiao’	Yunnan	Gui-Sheng Wu et al.2007
YNS015	DQ496386	Diannanxiao’	Yunnan	Gui-Sheng Wu et al.2007
YNS016	DQ496385	Diannanxiao’	Yunnan	Gui-Sheng Wu et al.2007
YNS017	DQ496384	Diannanxiao’	Yunnan	Gui-Sheng Wu et al.2007
YNS018	DQ496383	Diannanxiao’	Yunnan	Gui-Sheng Wu et al.2007
YNS019	DQ496382	Diannanxiao’	Yunnan	Gui-Sheng Wu et al.2007
YNS020	DQ496381	Diannanxiao’	Yunnan	Gui-Sheng Wu et al.2007
YNS021	DQ496380	Diannanxiao’	Yunnan	Gui-Sheng Wu et al.2007
YNS022	DQ496379	Diannanxiao’	Yunnan	Gui-Sheng Wu et al.2007
YNS023	DQ496378	Diannanxiao’	Yunnan	Gui-Sheng Wu et al.2007
YNS024	DQ496377	Diannanxiao’	Yunnan	Gui-Sheng Wu et al.2007
YNS025	DQ496376	Diannanxiao’	Yunnan	Gui-Sheng Wu et al.2007
YNS026	DQ496375	Diannanxiao’	Yunnan	Gui-Sheng Wu et al.2007
YNS027	DQ496374	Diannanxiao’	Yunnan	Gui-Sheng Wu et al.2007
YNS028	DQ496373	Diannanxiao’	Yunnan	Gui-Sheng Wu et al.2007
YNS029	DQ496372	Diannanxiao’	Yunnan	Gui-Sheng Wu et al.2007
YNS030	DQ496371	Diannanxiao’	Yunnan	Gui-Sheng Wu et al.2007
YNS031	DQ496370	Diannanxiao’	Yunnan	Gui-Sheng Wu et al.2007
YNS032	DQ496369	Diannanxiao’	Yunnan	Gui-Sheng Wu et al.2007
YNS033	DQ496368	Diannanxiao’	Yunnan	Gui-Sheng Wu et al.2007
YNS034	DQ496367	Diannanxiao’	Yunnan	Gui-Sheng Wu et al.2007
YNS035	DQ496366	Diannanxiao’	Yunnan	Gui-Sheng Wu et al.2007
YNS036	DQ496365	Diannanxiao’	Yunnan	Gui-Sheng Wu et al.2007
YNS037	DQ496308	Diannanxiao’	Yunnan	Gui-Sheng Wu et al.2007
YNS038	DQ496307	Diannanxiao’	Yunnan	Gui-Sheng Wu et al.2007
YNS039	DQ496306	Diannanxiao’	Yunnan	Gui-Sheng Wu et al.2007
YNS040	DQ496305	Diannanxiao’	Yunnan	Gui-Sheng Wu et al.2007
YNS041	DQ496304	Diannanxiao’	Yunnan	Gui-Sheng Wu et al.2007
YNS042	DQ496303	Diannanxiao’	Yunnan	Gui-Sheng Wu et al.2007
YNS043	HQ148516	Diannanxiao’	Yunnan,Ba	Gou,X et al. 2011
YNS044	HQ148515	Diannanxiao’	Yunnan,Ba	Gou,X et al. 2011
YNS045	HQ148514	Diannanxiao’	Yunnan,Ba	Gou,X et al. 2011
YNS046	HQ148513	Diannanxiao’	Yunnan,Ba	Gou,X et al. 2011
YNS047	HQ148512	Diannanxiao’	Yunnan,Ba	Gou,X et al. 2011
YNS048	HQ148511	Diannanxiao’	Yunnan,Ba	Gou,X et al. 2011

YNS049	HQ148510	Diannanxiao'	Yunnan,Ba	Gou,X et al. 2011
YNS050	HQ148509	Diannanxiao'	Yunnan,Ba	Gou,X et al. 2011
YNS051	HQ148508	Diannanxiao'	Yunnan,Ba	Gou,X et al. 2011
YNS052	HQ148507	Diannanxiao'	Yunnan,Ba	Gou,X et al. 2011
YNS053	HQ148506	Diannanxiao'	Yunnan,Ba	Gou,X et al. 2011
YNS054	HQ148505	Diannanxiao'	Yunnan,Ba	Gou,X et al. 2011
YNS055	HQ148504	Diannanxiao'	Yunnan,Ba	Gou,X et al. 2011
YNS056	HQ148503	Diannanxiao'	Yunnan,Ba	Gou,X et al. 2011
YNS057	HQ148502	Diannanxiao'	Yunnan,Ba	Gou,X et al. 2011
YNS058	HQ148501	Diannanxiao'	Yunnan,Ba	Gou,X et al. 2011
YNS059	HQ148500	Diannanxiao'	Yunnan,Ba	Gou,X et al. 2011
YNS060	HQ148499	Diannanxiao'	Yunnan,Ba	Gou,X et al. 2011
YNS061	HQ148498	Diannanxiao'	Yunnan,Ba	Gou,X et al. 2011
YNS062	HQ148497	Diannanxiao'	Yunnan,Ba	Gou,X et al. 2011
YNS063	HQ148496	Diannanxiao'	Yunnan,Ba	Gou,X et al. 2011
YNS064	HQ148495	Diannanxiao'	Yunnan,Ba	Gou,X et al. 2011
YNS065	HQ148494	Diannanxiao'	Yunnan,Ba	Gou,X et al. 2011
YNS066	HQ148493	Diannanxiao'	Yunnan,Ba	Gou,X et al. 2011
YNS067	HQ148492	Diannanxiao'	Yunnan,Ba	Gou,X et al. 2011
YNS068	HQ148491	Diannanxiao'	Yunnan,Ba	Gou,X et al. 2011
YNE001	EF545574	saba	YunnanSab	Gui-Sheng Wu et al.2007
YNE002	EF545567	saba	YunnanSab	Gui-Sheng Wu et al.2007
YNE003	DQ496692	Saba	Yunnan	Gui-Sheng Wu et al.2007
YNE004	DQ496691	Saba	Yunnan	Gui-Sheng Wu et al.2007
YNE005	DQ496690	Saba	Yunnan	Gui-Sheng Wu et al.2007
YNE006	DQ496689	Saba	Yunnan	Gui-Sheng Wu et al.2007
YNE007	DQ496688	Saba	Yunnan	Gui-Sheng Wu et al.2007
YNE008	DQ496687	Saba	Yunnan	Gui-Sheng Wu et al.2007
YNE009	DQ496686	Saba	Yunnan	Gui-Sheng Wu et al.2007
YNE010	DQ496685	Saba	Yunnan	Gui-Sheng Wu et al.2007
YNE011	DQ496684	Saba	Yunnan	Gui-Sheng Wu et al.2007
YNE012	DQ496683	Saba	Yunnan	Gui-Sheng Wu et al.2007
YNE013	DQ496682	Saba	Yunnan	Gui-Sheng Wu et al.2007
YNE014	DQ496681	Saba	Yunnan	Gui-Sheng Wu et al.2007
YNE015	DQ496680	Saba	Yunnan	Gui-Sheng Wu et al.2007
YNE016	DQ496679	Saba	Yunnan	Gui-Sheng Wu et al.2007
YNE017	DQ496678	Saba	Yunnan	Gui-Sheng Wu et al.2007
YNE018	DQ496677	Saba	Yunnan	Gui-Sheng Wu et al.2007
YNE019	DQ496676	Saba	Yunnan	Gui-Sheng Wu et al.2007
YNE020	DQ496675	Saba	Yunnan	Gui-Sheng Wu et al.2007
YNE021	DQ496674	Saba	Yunnan	Gui-Sheng Wu et al.2007
YNE022	DQ496673	Saba	Yunnan	Gui-Sheng Wu et al.2007
YNE023	DQ496672	Saba	Yunnan	Gui-Sheng Wu et al.2007
YNE024	DQ496671	Saba	Yunnan	Gui-Sheng Wu et al.2007
YNE025	DQ496670	Saba	Yunnan	Gui-Sheng Wu et al.2007
YNE026	DQ496669	Saba	Yunnan	Gui-Sheng Wu et al.2007
YNE027	DQ496668	Saba	Yunnan	Gui-Sheng Wu et al.2007
YNE028	DQ496667	Saba	Yunnan	Gui-Sheng Wu et al.2007
YNE029	DQ496666	Saba	Yunnan	Gui-Sheng Wu et al.2007
YNE030	DQ496665	Saba	Yunnan	Gui-Sheng Wu et al.2007
YNE031	DQ496664	Saba	Yunnan	Gui-Sheng Wu et al.2007
YNE032	DQ496663	Saba	Yunnan	Gui-Sheng Wu et al.2007
YNE033	DQ496662	Saba	Yunnan	Gui-Sheng Wu et al.2007
YNE034	DQ496661	Saba	Yunnan	Gui-Sheng Wu et al.2007
YNE035	DQ496660	Saba	Yunnan	Gui-Sheng Wu et al.2007
YNE036	DQ496659	Saba	Yunnan	Gui-Sheng Wu et al.2007
YNE037	DQ496658	Saba	Yunnan	Gui-Sheng Wu et al.2007
YNE038	DQ496657	Saba	Yunnan	Gui-Sheng Wu et al.2007

YNE039	DQ496656	Saba	Yunnan	Gui-Sheng Wu et al.2007
YNE040	DQ496655	Saba	Yunnan	Gui-Sheng Wu et al.2007
YNE041	HQ148490	Saba	Yunnan,Ch	Gou,X et al. 2011
YNE042	HQ148489	Saba	Yunnan,Ch	Gou,X et al. 2011
YNE043	HQ148488	Saba	Yunnan,Ch	Gou,X et al. 2011
YNE044	HQ148487	Saba	Yunnan,Ch	Gou,X et al. 2011
YNE045	HQ148486	Saba	Yunnan,Ch	Gou,X et al. 2011
YNE046	HQ148485	Saba	Yunnan,Ch	Gou,X et al. 2011
YNE047	HQ148484	Saba	Yunnan,Ch	Gou,X et al. 2011
YNE048	HQ148483	Saba	Yunnan,Ch	Gou,X et al. 2011
YNE049	HQ148482	Saba	Yunnan,Ch	Gou,X et al. 2011
YNE050	HQ148481	Saba	Yunnan,Ch	Gou,X et al. 2011
YNE051	HQ148480	Saba	Yunnan,Ch	Gou,X et al. 2011
YNE052	HQ148479	Saba	Yunnan,Ch	Gou,X et al. 2011
YNE053	HQ148478	Saba	Yunnan,Ch	Gou,X et al. 2011
YNE054	HQ148477	Saba	Yunnan,Ch	Gou,X et al. 2011
YNE055	HQ148476	Saba	Yunnan,Ch	Gou,X et al. 2011
YNE056	HQ148475	Saba	Yunnan,Ch	Gou,X et al. 2011
YNE057	HQ148474	Saba	Yunnan,Ch	Gou,X et al. 2011
YNE058	HQ148473	Saba	Yunnan,Ch	Gou,X et al. 2011
YNE059	HQ148472	Saba	Yunnan,Ch	Gou,X et al. 2011
YNE060	HQ148471	Saba	Yunnan,Ch	Gou,X et al. 2011
YNE061	HQ148470	Saba	Yunnan,Ch	Gou,X et al. 2011
YNE062	HQ148469	Saba	Yunnan,Ch	Gou,X et al. 2011
YNE063	HQ148468	Saba	Yunnan,Ch	Gou,X et al. 2011
YNE064	HQ148467	Saba	Yunnan,Ch	Gou,X et al. 2011
YNE065	HQ148466	Saba	Yunnan,Ch	Gou,X et al. 2011
YNE066	HQ148465	Saba	Yunnan,Ch	Gou,X et al. 2011
YNE067	HQ148464	Saba	Yunnan,Ch	Gou,X et al. 2011
YNE068	HQ148463	Saba	Yunnan,Ch	Gou,X et al. 2011
YNE069	HQ148462	Saba	Yunnan,Ch	Gou,X et al. 2011
YNE070	HQ148461	Saba	Yunnan,Ch	Gou,X et al. 2011
YNE071	HQ148460	Saba	Yunnan,Ch	Gou,X et al. 2011
YNE072	HQ148459	Saba	Yunnan,Ch	Gou,X et al. 2011
YNE073	HQ148458	Saba	Yunnan,Ch	Gou,X et al. 2011
YNE074	HQ148457	Saba	Yunnan,Ch	Gou,X et al. 2011
YNE075	HQ148456	Saba	Yunnan,Ch	Gou,X et al. 2011
YNE076	HQ148455	Saba	Yunnan,Ch	Gou,X et al. 2011
YNE077	HQ148454	Saba	Yunnan,Ch	Gou,X et al. 2011
YNE078	HQ148453	Saba	Yunnan,Ch	Gou,X et al. 2011
YNE079	HQ148452	Saba	Yunnan,Ch	Gou,X et al. 2011
YNE080	HQ148451	Saba	Yunnan,Ch	Gou,X et al. 2011
YNE081	HQ148450	Saba	Yunnan,Ch	Gou,X et al. 2011
YNE082	HQ148522	Wujin	Yunnan,Fu	Gou,X et al. 2011
YNE083	HQ148521	Wujin	Yunnan,Fu	Gou,X et al. 2011
YNE084	HQ148520	Wujin	Yunnan,Fu	Gou,X et al. 2011
YNE085	HQ148519	Wujin	Yunnan,Fu	Gou,X et al. 2011
YNE086	HQ148518	Wujin	Yunnan,Fu	Gou,X et al. 2011
YNE087	HQ148517	Wujin	Yunnan,Fu	Gou,X et al. 2011
YNE088	JX068485	Wujin	Sichuan	Jin,L et al.2012
YNE089	JX068484	Wujin	Sichuan	Jin,L et al.2012
YNE090	JX068483	Wujin	Sichuan	Jin,L et al.2012
YNE091	JX068482	Wujin	Sichuan	Jin,L et al.2012
YNE092	JX068481	Wujin	Sichuan	Jin,L et al.2012
YNE093	JX068480	Wujin	Sichuan	Jin,L et al.2012
YNE094	JX068479	Wujin	Sichuan	Jin,L et al.2012
YNE095	JX068478	Wujin	Sichuan	Jin,L et al.2012
YNE096	JX068477	Wujin	Sichuan	Jin,L et al.2012

YNE097	JX068476	Wujin	Sichuan	Jin,L et al.2012
YNE098	JX068475	Wujin	Sichuan	Jin,L et al.2012
YNE099	JX068474	Wujin	Sichuan	Jin,L et al.2012
YNE100	JX068473	Wujin	Sichuan	Jin,L et al.2012
YNE101	JX068472	Wujin	Sichuan	Jin,L et al.2012
YNE102	JX068471	Wujin	Sichuan	Jin,L et al.2012
YNE103	JX068470	Wujin	Sichuan	Jin,L et al.2012
YNE104	JX068469	Wujin	Sichuan	Jin,L et al.2012
YNE105	JX068468	Wujin	Sichuan	Jin,L et al.2012
YNE106	JX068467	Wujin	Sichuan	Jin,L et al.2012
YNE107	JX068466	Wujin	Sichuan	Jin,L et al.2012
YNE108	JX068465	Wujin	Sichuan	Jin,L et al.2012
YNE109	JX068464	Wujin	Sichuan	Jin,L et al.2012
YNE110	JX068463	Wujin	Sichuan	Jin,L et al.2012
YNE111	JX068462	Wujin	Sichuan	Jin,L et al.2012
YNE112	JX068461	Wujin	Sichuan	Jin,L et al.2012
YNE113	JX068460	Wujin	Sichuan	Jin,L et al.2012
YNE114	JX068459	Wujin	Sichuan	Jin,L et al.2012
YNE115	JX068458	Wujin	Sichuan	Jin,L et al.2012
YNE116	JX068457	Wujin	Sichuan	Jin,L et al.2012
YNE117	JX068456	Wujin	Sichuan	Jin,L et al.2012
YNE118	JX068455	Wujin	Sichuan	Jin,L et al.2012
YNE119	JX068454	Wujin	Sichuan	Jin,L et al.2012
YNE120	JX068453	Wujin	Sichuan	Jin,L et al.2012
YNE121	JX068452	Wujin	Sichuan	Jin,L et al.2012
YNE122	JX068451	Wujin	Sichuan	Jin,L et al.2012
YNE123	JX068450	Wujin	Sichuan	Jin,L et al.2012
YNE124	JX068449	Wujin	Sichuan	Jin,L et al.2012
YNE125	JX068448	Wujin	Sichuan	Jin,L et al.2012
YNE126	JX068447	Wujin	Sichuan	Jin,L et al.2012
YNE127	JX068446	Wujin	Sichuan	Jin,L et al.2012
YNE128	JX068445	Wujin	Sichuan	Jin,L et al.2012
SC001	JX068017	Chenghua	Sichuan	Jin,L et al.2012
SC002	JX068016	Chenghua	Sichuan	Jin,L et al.2012
SC003	JX068015	Chenghua	Sichuan	Jin,L et al.2012
SC004	JX068014	Chenghua	Sichuan	Jin,L et al.2012
SC005	JX068013	Chenghua	Sichuan	Jin,L et al.2012
SC006	JX068012	Chenghua	Sichuan	Jin,L et al.2012
SC007	DQ496338	Chenghua	Sichuan	Gui-Sheng Wu et al.2007
SC008	DQ496337	Chenghua	Sichuan	Gui-Sheng Wu et al.2007
SC009	DQ496336	Chenghua	Sichuan	Gui-Sheng Wu et al.2007
SC010	DQ496335	Chenghua	Sichuan	Gui-Sheng Wu et al.2007
SC011	EF590175	Chenghua	sichuan	Zhao, X
SC012	AF276929	Neijiang	sichuan	Kim, K. I et al. 2002
SC013	DQ379143.2	Neijiang	sichuan	Fang, M et al. 2006
SC014	DQ379142.2	Neijiang	sichuan	Fang, M et al. 2006
SC015	DQ379132.2	Neijiang	sichuan	Fang, M et al. 2006
SC016	DQ379131.2	Neijiang	sichuan	Fang, M et al. 2006
SC017	DQ152872.2	Neijiang	sichuan	Fang, M et al. 2006
SC018	JX068116	Neijiang	Sichuan	Jin,L et al.2012
SC019	JX068115	Neijiang	Sichuan	Jin,L et al.2012
SC020	JX068114	Neijiang	Sichuan	Jin,L et al.2012
SC021	JX068113	Neijiang	Sichuan	Jin,L et al.2012
SC022	JX068112	Neijiang	Sichuan	Jin,L et al.2012
SC023	JX068111	Neijiang	Sichuan	Jin,L et al.2012
SC024	JX068110	Neijiang	Sichuan	Jin,L et al.2012
SC025	JX068109	Neijiang	Sichuan	Jin,L et al.2012
SC026	JX068108	Neijiang	Sichuan	Jin,L et al.2012

SC027	JX068107	Neijiang	Sichuan	Jin,L et al.2012
SC028	JX068106	Neijiang	Sichuan	Jin,L et al.2012
SC029	JX068105	Neijiang	Sichuan	Jin,L et al.2012
SC030	JX068104	Neijiang	Sichuan	Jin,L et al.2012
SC031	JX068103	Neijiang	Sichuan	Jin,L et al.2012
SC032	JX068102	Neijiang	Sichuan	Jin,L et al.2012
SC033	JX068101	Neijiang	Sichuan	Jin,L et al.2012
SC034	JX068100	Neijiang	Sichuan	Jin,L et al.2012
SC035	JX068099	Neijiang	Sichuan	Jin,L et al.2012
SC036	JX068098	Neijiang	Sichuan	Jin,L et al.2012
SC037	JX068097	Neijiang	Sichuan	Jin,L et al.2012
SC038	JX068096	Neijiang	Sichuan	Jin,L et al.2012
SC039	JX068095	Neijiang	Sichuan	Jin,L et al.2012
SC040	JX068094	Neijiang	Sichuan	Jin,L et al.2012
SC041	JX068093	Neijiang	Sichuan	Jin,L et al.2012
SC042	JX068092	Neijiang	Sichuan	Jin,L et al.2012
SC043	JX068091	Neijiang	Sichuan	Jin,L et al.2012
SC044	JX068090	Neijiang	Sichuan	Jin,L et al.2012
SC045	JX068089	Neijiang	Sichuan	Jin,L et al.2012
SC046	JX068088	Neijiang	Sichuan	Jin,L et al.2012
SC047	JX068087	Neijiang	Sichuan	Jin,L et al.2012
SC048	JX068086	Neijiang	Sichuan	Jin,L et al.2012
SC049	JX068085	Neijiang	Sichuan	Jin,L et al.2012
SC050	JX068084	Neijiang	Sichuan	Jin,L et al.2012
SC051	JX068083	Neijiang	Sichuan	Jin,L et al.2012
SC052	JX068082	Neijiang	Sichuan	Jin,L et al.2012
SC053	JX068081	Neijiang	Sichuan	Jin,L et al.2012
SC054	JX068080	Neijiang	Sichuan	Jin,L et al.2012
SC055	JX068079	Neijiang	Sichuan	Jin,L et al.2012
SC056	JX068078	Neijiang	Sichuan	Jin,L et al.2012
SC057	JX068077	Neijiang	Sichuan	Jin,L et al.2012
SC058	JX068076	Neijiang	Sichuan	Jin,L et al.2012
SC059	JX068075	Neijiang	Sichuan	Jin,L et al.2012
SC060	JX068074	Neijiang	Sichuan	Jin,L et al.2012
SC061	JX068073	Neijiang	Sichuan	Jin,L et al.2012
SC062	JX068072	Neijiang	Sichuan	Jin,L et al.2012
SC063	JX068071	Neijiang	Sichuan	Jin,L et al.2012
SC064	JX068070	Neijiang	Sichuan	Jin,L et al.2012
SC065	JX068069	Neijiang	Sichuan	Jin,L et al.2012
SC066	JX068068	Neijiang	Sichuan	Jin,L et al.2012
SC067	DQ496596	Neijiang	sichuan	Gui-Sheng Wu et al.2007
SC068	DQ496595	Neijiang	sichuan	Gui-Sheng Wu et al.2007
SC069	DQ496594	Neijiang	sichuan	Gui-Sheng Wu et al.2007
SC070	DQ496593	Neijiang	sichuan	Gui-Sheng Wu et al.2007
SC071	DQ496592	Neijiang	sichuan	Gui-Sheng Wu et al.2007
SC072	DQ496591	Neijiang	sichuan	Gui-Sheng Wu et al.2007
SC073	DQ496590	Neijiang	sichuan	Gui-Sheng Wu et al.2007
SC074	DQ496589	Neijiang	sichuan	Gui-Sheng Wu et al.2007
SC075	DQ496588	Neijiang	sichuan	Gui-Sheng Wu et al.2007
SC076	DQ496587	Neijiang	sichuan	Gui-Sheng Wu et al.2007
SC077	DQ496586	Neijiang	sichuan	Gui-Sheng Wu et al.2007
SC078	DQ496585	Neijiang	sichuan	Gui-Sheng Wu et al.2007
SC079	DQ496584	Neijiang	sichuan	Gui-Sheng Wu et al.2007
SC080	DQ496583	Neijiang	sichuan	Gui-Sheng Wu et al.2007
SC081	DQ496582	Neijiang	sichuan	Gui-Sheng Wu et al.2007
SC082	HM026677	Neijiang	sichuan	Byeong-Woo Kim et al.2011
SC083	JX068439	Pengzhou	Sichuan	Jin,L et al.2012
SC084	JX068438	Pengzhou	Sichuan	Jin,L et al.2012

SC085	JX068437	Pengzhou	Sichuan	Jin,L et al.2012
SC086	JX068436	Pengzhou	Sichuan	Jin,L et al.2012
SC087	JX068435	Pengzhou	Sichuan	Jin,L et al.2012
SC088	JX068434	Pengzhou	Sichuan	Jin,L et al.2012
SC089	JX068433	Pengzhou	Sichuan	Jin,L et al.2012
SC090	JX068432	Pengzhou	Sichuan	Jin,L et al.2012
SC091	JX068431	Pengzhou	Sichuan	Jin,L et al.2012
SC092	JX068430	Pengzhou	Sichuan	Jin,L et al.2012
SC093	JX068429	Pengzhou	Sichuan	Jin,L et al.2012
SC094	JX068428	Pengzhou	Sichuan	Jin,L et al.2012
SC095	JX068427	Pengzhou	Sichuan	Jin,L et al.2012
SC096	JX068426	Pengzhou	Sichuan	Jin,L et al.2012
SC097	JX068425	Pengzhou	Sichuan	Jin,L et al.2012
SC098	JX068424	Pengzhou	Sichuan	Jin,L et al.2012
SC099	JX068423	Pengzhou	Sichuan	Jin,L et al.2012
SC100	JX068422	Pengzhou	Sichuan	Jin,L et al.2012
SC101	JX068421	Pengzhou	Sichuan	Jin,L et al.2012
SC102	JX068420	Pengzhou	Sichuan	Jin,L et al.2012
SC103	JX068419	Pengzhou	Sichuan	Jin,L et al.2012
SC104	JX068418	Pengzhou	Sichuan	Jin,L et al.2012
SC105	JX068417	Pengzhou	Sichuan	Jin,L et al.2012
SC106	JX068416	Pengzhou	Sichuan	Jin,L et al.2012
SC107	JX068415	Pengzhou	Sichuan	Jin,L et al.2012
SC108	JX068414	Pengzhou	Sichuan	Jin,L et al.2012
SC109	JX068413	Pengzhou	Sichuan	Jin,L et al.2012
SC110	JX068412	Pengzhou	Sichuan	Jin,L et al.2012
SC111	JX068411	Pengzhou	Sichuan	Jin,L et al.2012
SC112	JX068410	Pengzhou	Sichuan	Jin,L et al.2012
SC113	JX068409	Pengzhou	Sichuan	Jin,L et al.2012
SC114	JX068408	Pengzhou	Sichuan	Jin,L et al.2012
SC115	JX068407	Pengzhou	Sichuan	Jin,L et al.2012
SC116	JX068406	Pengzhou	Sichuan	Jin,L et al.2012
SC117	JX068405	Pengzhou	Sichuan	Jin,L et al.2012
SC118	JX068404	Pengzhou	Sichuan	Jin,L et al.2012
SC119	JX068403	Pengzhou	Sichuan	Jin,L et al.2012
SC120	JX068402	Pengzhou	Sichuan	Jin,L et al.2012
SC121	JX068401	Pengzhou	Sichuan	Jin,L et al.2012
SC122	JX068400	Pengzhou	Sichuan	Jin,L et al.2012
SC123	JX068399	Pengzhou	Sichuan	Jin,L et al.2012
SC124	JX068398	Pengzhou	Sichuan	Jin,L et al.2012
SC125	JX068397	Pengzhou	Sichuan	Jin,L et al.2012
SC126	JX068396	Pengzhou	Sichuan	Jin,L et al.2012
SC127	JX068395	Pengzhou	Sichuan	Jin,L et al.2012
SC128	JX068394	Pengzhou	Sichuan	Jin,L et al.2012
SC129	JX068393	Pengzhou	Sichuan	Jin,L et al.2012
SC130	JX068392	Pengzhou	Sichuan	Jin,L et al.2012
SC131	JX068391	Pengzhou	Sichuan	Jin,L et al.2012
SC133	DQ496653	Rongchang	Sichuan	Gui-Sheng Wu et al.2007
SC134	DQ496652	Rongchang	Sichuan	Gui-Sheng Wu et al.2007
SC135	DQ496651	Rongchang	Sichuan	Gui-Sheng Wu et al.2007
SC136	DQ496650	Rongchang	Sichuan	Gui-Sheng Wu et al.2007
SC137	DQ496649	Rongchang	Sichuan	Gui-Sheng Wu et al.2007
SC138	DQ496648	Rongchang	Sichuan	Gui-Sheng Wu et al.2007
SC139	DQ496647	Rongchang	Sichuan	Gui-Sheng Wu et al.2007
SC140	DQ496646	Rongchang	Sichuan	Gui-Sheng Wu et al.2007
SC141	DQ496645	Rongchang	Sichuan	Gui-Sheng Wu et al.2007
SC142	DQ496644	Rongchang	Sichuan	Gui-Sheng Wu et al.2007
SC143	HM026679	Rongchang	Sichuan	Byeong-Woo Kim et al.2011

SC144	DQ496975	Yahe	sichuan	Gui-Sheng Wu et al.2007
SC145	DQ496974	Yahe	sichuan	Gui-Sheng Wu et al.2007
SC146	DQ496973	Yahe	sichuan	Gui-Sheng Wu et al.2007
SC147	DQ496972	Yahe	sichuan	Gui-Sheng Wu et al.2007
SC148	DQ496971	Yahe	sichuan	Gui-Sheng Wu et al.2007
SC149	DQ496970	Yahe	sichuan	Gui-Sheng Wu et al.2007
SC150	JX068524	Yanan	Sichuan	Jin,L et al.2012
SC151	JX068523	Yanan	Sichuan	Jin,L et al.2012
SC152	JX068522	Yanan	Sichuan	Jin,L et al.2012
SC153	JX068521	Yanan	Sichuan	Jin,L et al.2012
SC154	JX068520	Yanan	Sichuan	Jin,L et al.2012
SC155	JX068519	Yanan	Sichuan	Jin,L et al.2012
SC156	JX068518	Yanan	Sichuan	Jin,L et al.2012
SC157	JX068517	Yanan	Sichuan	Jin,L et al.2012
SC158	JX068516	Yanan	Sichuan	Jin,L et al.2012
SC159	JX068515	Yanan	Sichuan	Jin,L et al.2012
SC160	JX068514	Yanan	Sichuan	Jin,L et al.2012
SC161	JX068513	Yanan	Sichuan	Jin,L et al.2012
SC162	JX068512	Yanan	Sichuan	Jin,L et al.2012
SC163	JX068511	Yanan	Sichuan	Jin,L et al.2012
SC164	JX068510	Yanan	Sichuan	Jin,L et al.2012
SC165	JX068509	Yanan	Sichuan	Jin,L et al.2012
SC166	JX068508	Yanan	Sichuan	Jin,L et al.2012
SC167	JX068507	Yanan	Sichuan	Jin,L et al.2012
SC168	JX068506	Yanan	Sichuan	Jin,L et al.2012
SC169	JX068505	Yanan	Sichuan	Jin,L et al.2012
SC170	JX068504	Yanan	Sichuan	Jin,L et al.2012
SC171	JX068503	Yanan	Sichuan	Jin,L et al.2012
SC172	JX068502	Yanan	Sichuan	Jin,L et al.2012
SC173	JX068501	Yanan	Sichuan	Jin,L et al.2012
SC174	JX068500	Yanan	Sichuan	Jin,L et al.2012
SC175	JX068499	Yanan	Sichuan	Jin,L et al.2012
SC176	JX068498	Yanan	Sichuan	Jin,L et al.2012
SC177	JX068497	Yanan	Sichuan	Jin,L et al.2012
SC178	JX068496	Yanan	Sichuan	Jin,L et al.2012
SC179	JX068495	Yanan	Sichuan	Jin,L et al.2012
SC180	JX068494	Yanan	Sichuan	Jin,L et al.2012
SC181	JX068493	Yanan	Sichuan	Jin,L et al.2012
SC182	JX068492	Yanan	Sichuan	Jin,L et al.2012
SC183	JX068491	Yanan	Sichuan	Jin,L et al.2012
SC184	JX068490	Yanan	Sichuan	Jin,L et al.2012
SC185	JX068489	Yanan	Sichuan	Jin,L et al.2012
SC186	JX068488	Yanan	Sichuan	Jin,L et al.2012
SC187	JX068487	Yanan	Sichuan	Jin,L et al.2012
SC188	JX068486	Yanan	Sichuan	Jin,L et al.2012
SC189	EF590200	Yanan	Sichuan	Zhao, X

CharacterizationoftheEuropeantypeofmaternallineageevidentinextantJejunativepigsJOURNALGene
MitochondrialdiversityinEuropeanandChinesepigsisconsistentwithpopulationexpansionsthatoccu
MitochondrialdiversityinEuropeanandChinesepigsisconsistentwithpopulationexpansionsthatoccu
MitochondrialdiversityinEuropeanandChinesepigsisconsistentwithpopulationexpansionsthatoccu
MitochondrialdiversityinEuropeanandChinesepigsisconsistentwithpopulationexpansionsthatoccu
MitochondrialdiversityinEuropeanandChinesepigsisconsistentwithpopulationexpansionsthatoccu
MitochondrialdiversityinEuropeanandChinesepigsisconsistentwithpopulationexpansionsthatoccu
MitochondrialdiversityinEuropeanandChinesepigsisconsistentwithpopulationexpansionsthatoccu
MitochondrialdiversityinEuropeanandChinesepigsisconsistentwithpopulationexpansionsthatoccu
MitochondrialdiversityinEuropeanandChinesepigsisconsistentwithpopulationexpansionsthatoccu
MitochondrialdiversityinEuropeanandChinesepigsisconsistentwithpopulationexpansionsthatoccu
PopulationphylogenomicanalysisofmitochondrialDNAinwildboarsanddomesticpigsrevealedmultiple
PopulationphylogenomicanalysisofmitochondrialDNAinwildboarsanddomesticpigsrevealedmultiple
PopulationphylogenomicanalysisofmitochondrialDNAinwildboarsanddomesticpigsrevealedmultiple
PopulationphylogenomicanalysisofmitochondrialDNAinwildboarsanddomesticpigsrevealedmultiple
PopulationphylogenomicanalysisofmitochondrialDNAinwildboarsanddomesticpigsrevealedmultiple
PopulationphylogenomicanalysisofmitochondrialDNAinwildboarsanddomesticpigsrevealedmultiple
PopulationphylogenomicanalysisofmitochondrialDNAinwildboarsanddomesticpigsrevealedmultiple
PopulationphylogenomicanalysisofmitochondrialDNAinwildboarsanddomesticpigsrevealedmultiple
PopulationphylogenomicanalysisofmitochondrialDNAinwildboarsanddomesticpigsrevealedmultiple
PopulationphylogenomicanalysisofmitochondrialDNAinwildboarsanddomesticpigsrevealedmultiple
PopulationphylogenomicanalysisofmitochondrialDNAinwildboarsanddomesticpigsrevealedmultiple
PopulationphylogenomicanalysisofmitochondrialDNAinwildboarsanddomesticpigsrevealedmultiple
PopulationphylogenomicanalysisofmitochondrialDNAinwildboarsanddomesticpigsrevealedmultiple
PopulationphylogenomicanalysisofmitochondrialDNAinwildboarsanddomesticpigsrevealedmultiple
PopulationphylogenomicanalysisofmitochondrialDNAinwildboarsanddomesticpigsrevealedmultiple
PopulationphylogenomicanalysisofmitochondrialDNAinwildboarsanddomesticpigsrevealedmultiple
PopulationphylogenomicanalysisofmitochondrialDNAinwildboarsanddomesticpigsrevealedmultiple
PopulationphylogenomicanalysisofmitochondrialDNAinwildboarsanddomesticpigsrevealedmultiple
PopulationphylogenomicanalysisofmitochondrialDNAinwildboarsanddomesticpigsrevealedmultiple
PopulationphylogenomicanalysisofmitochondrialDNAinwildboarsanddomesticpigsrevealedmultiple
PopulationphylogenomicanalysisofmitochondrialDNAinwildboarsanddomesticpigsrevealedmultiple
PopulationphylogenomicanalysisofmitochondrialDNAinwildboarsanddomesticpigsrevealedmultiple
PopulationphylogenomicanalysisofmitochondrialDNAinwildboarsanddomesticpigsrevealedmultiple
PopulationphylogenomicanalysisofmitochondrialDNAinwildboarsanddomesticpigsrevealedmultiple
PopulationphylogenomicanalysisofmitochondrialDNAinwildboarsanddomesticpigsrevealedmultiple
PopulationphylogenomicanalysisofmitochondrialDNAinwildboarsanddomesticpigsrevealedmultiple
PopulationphylogenomicanalysisofmitochondrialDNAinwildboarsanddomesticpigsrevealedmultiple
PopulationphylogenomicanalysisofmitochondrialDNAinwildboarsanddomesticpigsrevealedmultiple
PopulationphylogenomicanalysisofmitochondrialDNAinwildboarsanddomesticpigsrevealedmultiple
This study
This study
This study
This study
This study
This study
This study
This study
This study
This study
This study
This study
This study

This study
This study
This study
This study
This study
This study
This study
This study
This study
This study
This study
This study
This study
This study
This study
This study
This study
This study
This study
This study
This study
This study
This study
This study
This study
This study
This study
This study
This study
This study
This study
This study
This study
This study
This study
This study
This study
This study
This study
This study
This study
This study
This study
This study
This study
This study
This study
This study
This study
This study
This study
This study
This study
This study
This study

This study
This study
This study
This study
This study
Populationphylogenomicanalysisofmitochondrial DNAinwildboarsanddomesticpigsrevealedmultiple
Populationphylogenomicanalysisofmitochondrial DNAinwildboarsanddomesticpigsrevealedmultiple
Populationphylogenomicanalysisofmitochondrial DNAinwildboarsanddomesticpigsrevealedmultiple
Populationphylogenomicanalysisofmitochondrial DNAinwildboarsanddomesticpigsrevealedmultiple
Populationphylogenomicanalysisofmitochondrial DNAinwildboarsanddomesticpigsrevealedmultiple
Populationphylogenomicanalysisofmitochondrial DNAinwildboarsanddomesticpigsrevealedmultiple
Populationphylogenomicanalysisofmitochondrial DNAinwildboarsanddomesticpigsrevealedmultiple
Populationphylogenomicanalysisofmitochondrial DNAinwildboarsanddomesticpigsrevealedmultiple
Populationphylogenomicanalysisofmitochondrial DNAinwildboarsanddomesticpigsrevealedmultiple
Populationphylogenomicanalysisofmitochondrial DNAinwildboarsanddomesticpigsrevealedmultiple
Populationphylogenomicanalysisofmitochondrial DNAinwildboarsanddomesticpigsrevealedmultiple
Populationphylogenomicanalysisofmitochondrial DNAinwildboarsanddomesticpigsrevealedmultiple
Populationphylogenomicanalysisofmitochondrial DNAinwildboarsanddomesticpigsrevealedmultiple
Populationphylogenomicanalysisofmitochondrial DNAinwildboarsanddomesticpigsrevealedmultiple
Populationphylogenomicanalysisofmitochondrial DNAinwildboarsanddomesticpigsrevealedmultiple
Populationphylogenomicanalysisofmitochondrial DNAinwildboarsanddomesticpigsrevealedmultiple
Populationphylogenomicanalysisofmitochondrial DNAinwildboarsanddomesticpigsrevealedmultiple
Populationphylogenomicanalysisofmitochondrial DNAinwildboarsanddomesticpigsrevealedmultiple
Populationphylogenomicanalysisofmitochondrial DNAinwildboarsanddomesticpigsrevealedmultiple
ThelocaloriginofthetibetanpigandadditionalinsightsintotheoriginofAsianpigsJOURNALPLoSONE6
ThelocaloriginofthetibetanpigandadditionalinsightsintotheoriginofAsianpigsJOURNALPLoSONE6
ThelocaloriginofthetibetanpigandadditionalinsightsintotheoriginofAsianpigsJOURNALPLoSONE6
ThelocaloriginofthetibetanpigandadditionalinsightsintotheoriginofAsianpigsJOURNALPLoSONE6
ThelocaloriginofthetibetanpigandadditionalinsightsintotheoriginofAsianpigsJOURNALPLoSONE6
ThelocaloriginofthetibetanpigandadditionalinsightsintotheoriginofAsianpigsJOURNALPLoSONE6
ThelocaloriginofthetibetanpigandadditionalinsightsintotheoriginofAsianpigsJOURNALPLoSONE6
ThelocaloriginofthetibetanpigandadditionalinsightsintotheoriginofAsianpigsJOURNALPLoSONE6
ThelocaloriginofthetibetanpigandadditionalinsightsintotheoriginofAsianpigsJOURNALPLoSONE6
ThelocaloriginofthetibetanpigandadditionalinsightsintotheoriginofAsianpigsJOURNALPLoSONE6
ThelocaloriginofthetibetanpigandadditionalinsightsintotheoriginofAsianpigsJOURNALPLoSONE6
ThelocaloriginofthetibetanpigandadditionalinsightsintotheoriginofAsianpigsJOURNALPLoSONE6
ThelocaloriginofthetibetanpigandadditionalinsightsintotheoriginofAsianpigsJOURNALPLoSONE6
ThelocaloriginofthetibetanpigandadditionalinsightsintotheoriginofAsianpigsJOURNALPLoSONE6
ThelocaloriginofthetibetanpigandadditionalinsightsintotheoriginofAsianpigsJOURNALPLoSONE6
ThelocaloriginofthetibetanpigandadditionalinsightsintotheoriginofAsianpigsJOURNALPLoSONE6
ThelocaloriginofthetibetanpigandadditionalinsightsintotheoriginofAsianpigsJOURNALPLoSONE6
ThelocaloriginofthetibetanpigandadditionalinsightsintotheoriginofAsianpigsJOURNALPLoSONE6
Populationphylogenomicanalysisofmitochondrial DNAinwildboarsanddomesticpigsrevealedmultiple
Populationphylogenomicanalysisofmitochondrial DNAinwildboarsanddomesticpigsrevealedmultiple
Populationphylogenomicanalysisofmitochondrial DNAinwildboarsanddomesticpigsrevealedmultiple
Populationphylogenomicanalysisofmitochondrial DNAinwildboarsanddomesticpigsrevealedmultiple
Populationphylogenomicanalysisofmitochondrial DNAinwildboarsanddomesticpigsrevealedmultiple
Populationphylogenomicanalysisofmitochondrial DNAinwildboarsanddomesticpigsrevealedmultiple
Populationphylogenomicanalysisofmitochondrial DNAinwildboarsanddomesticpigsrevealedmultiple
Populationphylogenomicanalysisofmitochondrial DNAinwildboarsanddomesticpigsrevealedmultiple
Populationphylogenomicanalysisofmitochondrial DNAinwildboarsanddomesticpigsrevealedmultiple
Populationphylogenomicanalysisofmitochondrial DNAinwildboarsanddomesticpigsrevealedmultiple
Populationphylogenomicanalysisofmitochondrial DNAinwildboarsanddomesticpigsrevealedmultiple
Populationphylogenomicanalysisofmitochondrial DNAinwildboarsanddomesticpigsrevealedmultiple
Populationphylogenomicanalysisofmitochondrial DNAinwildboarsanddomesticpigsrevealedmultiple
Populationphylogenomicanalysisofmitochondrial DNAinwildboarsanddomesticpigsrevealedmultiple
Populationphylogenomicanalysisofmitochondrial DNAinwildboarsanddomesticpigsrevealedmultiple

PopulationphylogenomicanalysisofmitochondrialDNAinwildboarsanddomesticpigsrevealedmultiple
ThelocaloriginofthetibetanpigandadditionalinsightsintotheoriginofAsianpigsJOURNALPLoSONE6
ThelocaloriginofthetibetanpigandadditionalinsightsintotheoriginofAsianpigsJOURNALPLoSONE6
ThelocaloriginofthetibetanpigandadditionalinsightsintotheoriginofAsianpigsJOURNALPLoSONE6
ThelocaloriginofthetibetanpigandadditionalinsightsintotheoriginofAsianpigsJOURNALPLoSONE6
ThelocaloriginofthetibetanpigandadditionalinsightsintotheoriginofAsianpigsJOURNALPLoSONE6
ThelocaloriginofthetibetanpigandadditionalinsightsintotheoriginofAsianpigsJOURNALPLoSONE6
ThelocaloriginofthetibetanpigandadditionalinsightsintotheoriginofAsianpigsJOURNALPLoSONE6
ThelocaloriginofthetibetanpigandadditionalinsightsintotheoriginofAsianpigsJOURNALPLoSONE6
ThelocaloriginofthetibetanpigandadditionalinsightsintotheoriginofAsianpigsJOURNALPLoSONE6
ThelocaloriginofthetibetanpigandadditionalinsightsintotheoriginofAsianpigsJOURNALPLoSONE6
Complete sequence and geneorganization of the Dahe pig mitochondrial genome
PopulationphylogenomicanalysisofmitochondrialDNAinwildboarsanddomesticpigsrevealedmultiple
PopulationphylogenomicanalysisofmitochondrialDNAinwildboarsanddomesticpigsrevealedmultiple
PopulationphylogenomicanalysisofmitochondrialDNAinwildboarsanddomesticpigsrevealedmultiple
PopulationphylogenomicanalysisofmitochondrialDNAinwildboarsanddomesticpigsrevealedmultiple
PopulationphylogenomicanalysisofmitochondrialDNAinwildboarsanddomesticpigsrevealedmultiple
PopulationphylogenomicanalysisofmitochondrialDNAinwildboarsanddomesticpigsrevealedmultiple
PopulationphylogenomicanalysisofmitochondrialDNAinwildboarsanddomesticpigsrevealedmultiple
PopulationphylogenomicanalysisofmitochondrialDNAinwildboarsanddomesticpigsrevealedmultiple
PopulationphylogenomicanalysisofmitochondrialDNAinwildboarsanddomesticpigsrevealedmultiple
PopulationphylogenomicanalysisofmitochondrialDNAinwildboarsanddomesticpigsrevealedmultiple
PopulationphylogenomicanalysisofmitochondrialDNAinwildboarsanddomesticpigsrevealedmultiple
PopulationphylogenomicanalysisofmitochondrialDNAinwildboarsanddomesticpigsrevealedmultiple
PopulationphylogenomicanalysisofmitochondrialDNAinwildboarsanddomesticpigsrevealedmultiple
PopulationphylogenomicanalysisofmitochondrialDNAinwildboarsanddomesticpigsrevealedmultiple
PopulationphylogenomicanalysisofmitochondrialDNAinwildboarsanddomesticpigsrevealedmultiple
PopulationphylogenomicanalysisofmitochondrialDNAinwildboarsanddomesticpigsrevealedmultiple
PopulationphylogenomicanalysisofmitochondrialDNAinwildboarsanddomesticpigsrevealedmultiple
PopulationphylogenomicanalysisofmitochondrialDNAinwildboarsanddomesticpigsrevealedmultiple
PopulationphylogenomicanalysisofmitochondrialDNAinwildboarsanddomesticpigsrevealedmultiple
PopulationphylogenomicanalysisofmitochondrialDNAinwildboarsanddomesticpigsrevealedmultiple
PopulationphylogenomicanalysisofmitochondrialDNAinwildboarsanddomesticpigsrevealedmultiple
PopulationphylogenomicanalysisofmitochondrialDNAinwildboarsanddomesticpigsrevealedmultiple
PopulationphylogenomicanalysisofmitochondrialDNAinwildboarsanddomesticpigsrevealedmultiple
PopulationphylogenomicanalysisofmitochondrialDNAinwildboarsanddomesticpigsrevealedmultiple
PopulationphylogenomicanalysisofmitochondrialDNAinwildboarsanddomesticpigsrevealedmultiple
PopulationphylogenomicanalysisofmitochondrialDNAinwildboarsanddomesticpigsrevealedmultiple
PopulationphylogenomicanalysisofmitochondrialDNAinwildboarsanddomesticpigsrevealedmultiple
PopulationphylogenomicanalysisofmitochondrialDNAinwildboarsanddomesticpigsrevealedmultiple
PopulationphylogenomicanalysisofmitochondrialDNAinwildboarsanddomesticpigsrevealedmultiple
PopulationphylogenomicanalysisofmitochondrialDNAinwildboarsanddomesticpigsrevealedmultiple
PopulationphylogenomicanalysisofmitochondrialDNAinwildboarsanddomesticpigsrevealedmultiple
PopulationphylogenomicanalysisofmitochondrialDNAinwildboarsanddomesticpigsrevealedmultiple
PopulationphylogenomicanalysisofmitochondrialDNAinwildboarsanddomesticpigsrevealedmultiple
PopulationphylogenomicanalysisofmitochondrialDNAinwildboarsanddomesticpigsrevealedmultiple
PopulationphylogenomicanalysisofmitochondrialDNAinwildboarsanddomesticpigsrevealedmultiple
PopulationphylogenomicanalysisofmitochondrialDNAinwildboarsanddomesticpigsrevealedmultiple
PopulationphylogenomicanalysisofmitochondrialDNAinwildboarsanddomesticpigsrevealedmultiple
PopulationphylogenomicanalysisofmitochondrialDNAinwildboarsanddomesticpigsrevealedmultiple
PopulationphylogenomicanalysisofmitochondrialDNAinwildboarsanddomesticpigsrevealedmultiple
PopulationphylogenomicanalysisofmitochondrialDNAinwildboarsanddomesticpigsrevealedmultiple
PopulationphylogenomicanalysisofmitochondrialDNAinwildboarsanddomesticpigsrevealedmultiple
PopulationphylogenomicanalysisofmitochondrialDNAinwildboarsanddomesticpigsrevealedmultiple
PopulationphylogenomicanalysisofmitochondrialDNAinwildboarsanddomesticpigsrevealedmultiple
PopulationphylogenomicanalysisofmitochondrialDNAinwildboarsanddomesticpigsrevealedmultiple
PopulationphylogenomicanalysisofmitochondrialDNAinwildboarsanddomesticpigsrevealedmultiple
ThelocaloriginofthetibetanpigandadditionalinsightsintotheoriginofAsianpigsJOURNALPLoSONE6
ThelocaloriginofthetibetanpigandadditionalinsightsintotheoriginofAsianpigsJOURNALPLoSONE6
ThelocaloriginofthetibetanpigandadditionalinsightsintotheoriginofAsianpigsJOURNALPLoSONE6
ThelocaloriginofthetibetanpigandadditionalinsightsintotheoriginofAsianpigsJOURNALPLoSONE6
ThelocaloriginofthetibetanpigandadditionalinsightsintotheoriginofAsianpigsJOURNALPLoSONE6
ThelocaloriginofthetibetanpigandadditionalinsightsintotheoriginofAsianpigsJOURNALPLoSONE6

ThelocaloriginofthetibetanpigandadditionalinsightsintotheoriginofAsianpigsJOURNALPLoSONE6
ThelocaloriginofthetibetanpigandadditionalinsightsintotheoriginofAsianpigsJOURNALPLoSONE6
ThelocaloriginofthetibetanpigandadditionalinsightsintotheoriginofAsianpigsJOURNALPLoSONE6
ThelocaloriginofthetibetanpigandadditionalinsightsintotheoriginofAsianpigsJOURNALPLoSONE6
ThelocaloriginofthetibetanpigandadditionalinsightsintotheoriginofAsianpigsJOURNALPLoSONE6
ThelocaloriginofthetibetanpigandadditionalinsightsintotheoriginofAsianpigsJOURNALPLoSONE6
ThelocaloriginofthetibetanpigandadditionalinsightsintotheoriginofAsianpigsJOURNALPLoSONE6
ThelocaloriginofthetibetanpigandadditionalinsightsintotheoriginofAsianpigsJOURNALPLoSONE6
ThelocaloriginofthetibetanpigandadditionalinsightsintotheoriginofAsianpigsJOURNALPLoSONE6
ThelocaloriginofthetibetanpigandadditionalinsightsintotheoriginofAsianpigsJOURNALPLoSONE6
ThelocaloriginofthetibetanpigandadditionalinsightsintotheoriginofAsianpigsJOURNALPLoSONE6
ThelocaloriginofthetibetanpigandadditionalinsightsintotheoriginofAsianpigsJOURNALPLoSONE6
ThelocaloriginofthetibetanpigandadditionalinsightsintotheoriginofAsianpigsJOURNALPLoSONE6
ThelocaloriginofthetibetanpigandadditionalinsightsintotheoriginofAsianpigsJOURNALPLoSONE6
ThelocaloriginofthetibetanpigandadditionalinsightsintotheoriginofAsianpigsJOURNALPLoSONE6
ThelocaloriginofthetibetanpigandadditionalinsightsintotheoriginofAsianpigsJOURNALPLoSONE6
ThelocaloriginofthetibetanpigandadditionalinsightsintotheoriginofAsianpigsJOURNALPLoSONE6
ThelocaloriginofthetibetanpigandadditionalinsightsintotheoriginofAsianpigsJOURNALPLoSONE6
ThelocaloriginofthetibetanpigandadditionalinsightsintotheoriginofAsianpigsJOURNALPLoSONE6
ThelocaloriginofthetibetanpigandadditionalinsightsintotheoriginofAsianpigsJOURNALPLoSONE6
PopulationphylogenomicanalysisofmitochondrialDNAinwildboarsanddomesticpigsrevealedmultiple
PopulationphylogenomicanalysisofmitochondrialDNAinwildboarsanddomesticpigsrevealedmultiple
PopulationphylogenomicanalysisofmitochondrialDNAinwildboarsanddomesticpigsrevealedmultiple
PopulationphylogenomicanalysisofmitochondrialDNAinwildboarsanddomesticpigsrevealedmultiple
PopulationphylogenomicanalysisofmitochondrialDNAinwildboarsanddomesticpigsrevealedmultiple
PopulationphylogenomicanalysisofmitochondrialDNAinwildboarsanddomesticpigsrevealedmultiple
PopulationphylogenomicanalysisofmitochondrialDNAinwildboarsanddomesticpigsrevealedmultiple
PopulationphylogenomicanalysisofmitochondrialDNAinwildboarsanddomesticpigsrevealedmultiple
PopulationphylogenomicanalysisofmitochondrialDNAinwildboarsanddomesticpigsrevealedmultiple
PopulationphylogenomicanalysisofmitochondrialDNAinwildboarsanddomesticpigsrevealedmultiple
PopulationphylogenomicanalysisofmitochondrialDNAinwildboarsanddomesticpigsrevealedmultiple
PopulationphylogenomicanalysisofmitochondrialDNAinwildboarsanddomesticpigsrevealedmultiple
PopulationphylogenomicanalysisofmitochondrialDNAinwildboarsanddomesticpigsrevealedmultiple
PopulationphylogenomicanalysisofmitochondrialDNAinwildboarsanddomesticpigsrevealedmultiple
PopulationphylogenomicanalysisofmitochondrialDNAinwildboarsanddomesticpigsrevealedmultiple
PopulationphylogenomicanalysisofmitochondrialDNAinwildboarsanddomesticpigsrevealedmultiple
PopulationphylogenomicanalysisofmitochondrialDNAinwildboarsanddomesticpigsrevealedmultiple
PopulationphylogenomicanalysisofmitochondrialDNAinwildboarsanddomesticpigsrevealedmultiple
PopulationphylogenomicanalysisofmitochondrialDNAinwildboarsanddomesticpigsrevealedmultiple
PopulationphylogenomicanalysisofmitochondrialDNAinwildboarsanddomesticpigsrevealedmultiple
PopulationphylogenomicanalysisofmitochondrialDNAinwildboarsanddomesticpigsrevealedmultiple
PopulationphylogenomicanalysisofmitochondrialDNAinwildboarsanddomesticpigsrevealedmultiple
PopulationphylogenomicanalysisofmitochondrialDNAinwildboarsanddomesticpigsrevealedmultiple
PopulationphylogenomicanalysisofmitochondrialDNAinwildboarsanddomesticpigsrevealedmultiple
PopulationphylogenomicanalysisofmitochondrialDNAinwildboarsanddomesticpigsrevealedmultiple
PopulationphylogenomicanalysisofmitochondrialDNAinwildboarsanddomesticpigsrevealedmultiple
PopulationphylogenomicanalysisofmitochondrialDNAinwildboarsanddomesticpigsrevealedmultiple
PopulationphylogenomicanalysisofmitochondrialDNAinwildboarsanddomesticpigsrevealedmultiple
PopulationphylogenomicanalysisofmitochondrialDNAinwildboarsanddomesticpigsrevealedmultiple
PopulationphylogenomicanalysisofmitochondrialDNAinwildboarsanddomesticpigsrevealedmultiple
PopulationphylogenomicanalysisofmitochondrialDNAinwildboarsanddomesticpigsrevealedmultiple
PopulationphylogenomicanalysisofmitochondrialDNAinwildboarsanddomesticpigsrevealedmultiple
PopulationphylogenomicanalysisofmitochondrialDNAinwildboarsanddomesticpigsrevealedmultiple
PopulationphylogenomicanalysisofmitochondrialDNAinwildboarsanddomesticpigsrevealedmultiple
PopulationphylogenomicanalysisofmitochondrialDNAinwildboarsanddomesticpigsrevealedmultiple
PopulationphylogenomicanalysisofmitochondrialDNAinwildboarsanddomesticpigsrevealedmultiple
PopulationphylogenomicanalysisofmitochondrialDNAinwildboarsanddomesticpigsrevealedmultiple
PopulationphylogenomicanalysisofmitochondrialDNAinwildboarsanddomesticpigsrevealedmultiple
PopulationphylogenomicanalysisofmitochondrialDNAinwildboarsanddomesticpigsrevealedmultiple

Populationphylogenomicanalysisofmitochondrial DNAinwildboarsanddomesticpigsrevealedmultipl
Populationphylogenomicanalysisofmitochondrial DNAinwildboarsanddomesticpigsrevealedmultipl
ThelocaloriginofthetibetanpigandadditionalinsightsintotheoriginofAsianpigsJOURNALPLoSONE6
ThelocaloriginofthetibetanpigandadditionalinsightsintotheoriginofAsianpigsJOURNALPLoSONE6
ThelocaloriginofthetibetanpigandadditionalinsightsintotheoriginofAsianpigsJOURNALPLoSONE6
ThelocaloriginofthetibetanpigandadditionalinsightsintotheoriginofAsianpigsJOURNALPLoSONE6
ThelocaloriginofthetibetanpigandadditionalinsightsintotheoriginofAsianpigsJOURNALPLoSONE6
ThelocaloriginofthetibetanpigandadditionalinsightsintotheoriginofAsianpigsJOURNALPLoSONE6
ThelocaloriginofthetibetanpigandadditionalinsightsintotheoriginofAsianpigsJOURNALPLoSONE6
ThelocaloriginofthetibetanpigandadditionalinsightsintotheoriginofAsianpigsJOURNALPLoSONE6
ThelocaloriginofthetibetanpigandadditionalinsightsintotheoriginofAsianpigsJOURNALPLoSONE6
ThelocaloriginofthetibetanpigandadditionalinsightsintotheoriginofAsianpigsJOURNALPLoSONE6
ThelocaloriginofthetibetanpigandadditionalinsightsintotheoriginofAsianpigsJOURNALPLoSONE6
ThelocaloriginofthetibetanpigandadditionalinsightsintotheoriginofAsianpigsJOURNALPLoSONE6
ThelocaloriginofthetibetanpigandadditionalinsightsintotheoriginofAsianpigsJOURNALPLoSONE6
ThelocaloriginofthetibetanpigandadditionalinsightsintotheoriginofAsianpigsJOURNALPLoSONE6
ThelocaloriginofthetibetanpigandadditionalinsightsintotheoriginofAsianpigsJOURNALPLoSONE6
ThelocaloriginofthetibetanpigandadditionalinsightsintotheoriginofAsianpigsJOURNALPLoSONE6
ThelocaloriginofthetibetanpigandadditionalinsightsintotheoriginofAsianpigsJOURNALPLoSONE6
ThelocaloriginofthetibetanpigandadditionalinsightsintotheoriginofAsianpigsJOURNALPLoSONE6
ThelocaloriginofthetibetanpigandadditionalinsightsintotheoriginofAsianpigsJOURNALPLoSONE6
ThelocaloriginofthetibetanpigandadditionalinsightsintotheoriginofAsianpigsJOURNALPLoSONE6
ThelocaloriginofthetibetanpigandadditionalinsightsintotheoriginofAsianpigsJOURNALPLoSONE6
ThelocaloriginofthetibetanpigandadditionalinsightsintotheoriginofAsianpigsJOURNALPLoSONE6
ThelocaloriginofthetibetanpigandadditionalinsightsintotheoriginofAsianpigsJOURNALPLoSONE6
ThelocaloriginofthetibetanpigandadditionalinsightsintotheoriginofAsianpigsJOURNALPLoSONE6
ThelocaloriginofthetibetanpigandadditionalinsightsintotheoriginofAsianpigsJOURNALPLoSONE6
ThelocaloriginofthetibetanpigandadditionalinsightsintotheoriginofAsianpigsJOURNALPLoSONE6
ThelocaloriginofthetibetanpigandadditionalinsightsintotheoriginofAsianpigsJOURNALPLoSONE6
ThelocaloriginofthetibetanpigandadditionalinsightsintotheoriginofAsianpigsJOURNALPLoSONE6
ThelocaloriginofthetibetanpigandadditionalinsightsintotheoriginofAsianpigsJOURNALPLoSONE6
ThelocaloriginofthetibetanpigandadditionalinsightsintotheoriginofAsianpigsJOURNALPLoSONE6
ThelocaloriginofthetibetanpigandadditionalinsightsintotheoriginofAsianpigsJOURNALPLoSONE6
ThelocaloriginofthetibetanpigandadditionalinsightsintotheoriginofAsianpigsJOURNALPLoSONE6
ThelocaloriginofthetibetanpigandadditionalinsightsintotheoriginofAsianpigsJOURNALPLoSONE6
ThelocaloriginofthetibetanpigandadditionalinsightsintotheoriginofAsianpigsJOURNALPLoSONE6
ThelocaloriginofthetibetanpigandadditionalinsightsintotheoriginofAsianpigsJOURNALPLoSONE6
ThelocaloriginofthetibetanpigandadditionalinsightsintotheoriginofAsianpigsJOURNALPLoSONE6
ThelocaloriginofthetibetanpigandadditionalinsightsintotheoriginofAsianpigsJOURNALPLoSONE6
ThelocaloriginofthetibetanpigandadditionalinsightsintotheoriginofAsianpigsJOURNALPLoSONE6
ThelocaloriginofthetibetanpigandadditionalinsightsintotheoriginofAsianpigsJOURNALPLoSONE6
ThelocaloriginofthetibetanpigandadditionalinsightsintotheoriginofAsianpigsJOURNALPLoSONE6
ThelocaloriginofthetibetanpigandadditionalinsightsintotheoriginofAsianpigsJOURNALPLoSONE6
ThelocaloriginofthetibetanpigandadditionalinsightsintotheoriginofAsianpigsJOURNALPLoSONE6
ThelocaloriginofthetibetanpigandadditionalinsightsintotheoriginofAsianpigsJOURNALPLoSONE6
ThelocaloriginofthetibetanpigandadditionalinsightsintotheoriginofAsianpigsJOURNALPLoSONE6
ThelocaloriginofthetibetanpigandadditionalinsightsintotheoriginofAsianpigsJOURNALPLoSONE6
ThelocaloriginofthetibetanpigandadditionalinsightsintotheoriginofAsianpigsJOURNALPLoSONE6
ThelocaloriginofthetibetanpigandadditionalinsightsintotheoriginofAsianpigsJOURNALPLoSONE6
MitochondrialDNAevidenceindicatesthelocaloriginofdomesticpigsintheupstreamregionoftheyang
MitochondrialDNAevidenceindicatesthelocaloriginofdomesticpigsintheupstreamregionoftheyang
MitochondrialDNAevidenceindicatesthelocaloriginofdomesticpigsintheupstreamregionoftheyang
MitochondrialDNAevidenceindicatesthelocaloriginofdomesticpigsintheupstreamregionoftheyang
MitochondrialDNAevidenceindicatesthelocaloriginofdomesticpigsintheupstreamregionoftheyang
MitochondrialDNAevidenceindicatesthelocaloriginofdomesticpigsintheupstreamregionoftheyang
MitochondrialDNAevidenceindicatesthelocaloriginofdomesticpigsintheupstreamregionoftheyang
MitochondrialDNAevidenceindicatesthelocaloriginofdomesticpigsintheupstreamregionoftheyang
MitochondrialDNAevidenceindicatesthelocaloriginofdomesticpigsintheupstreamregionoftheyang

MitochondrialDNAevidenceindicatesthelocaloriginofdomesticpigsintheupstreamregionoftheyang
MitochondrialDNAevidenceindicatesthelocaloriginofdomesticpigsintheupstreamregionoftheyang
MitochondrialDNAevidenceindicatesthelocaloriginofdomesticpigsintheupstreamregionoftheyang
MitochondrialDNAevidenceindicatesthelocaloriginofdomesticpigsintheupstreamregionoftheyang
MitochondrialDNAevidenceindicatesthelocaloriginofdomesticpigsintheupstreamregionoftheyang
MitochondrialDNAevidenceindicatesthelocaloriginofdomesticpigsintheupstreamregionoftheyang
MitochondrialDNAevidenceindicatesthelocaloriginofdomesticpigsintheupstreamregionoftheyang
MitochondrialDNAevidenceindicatesthelocaloriginofdomesticpigsintheupstreamregionoftheyang
MitochondrialDNAevidenceindicatesthelocaloriginofdomesticpigsintheupstreamregionoftheyang
MitochondrialDNAevidenceindicatesthelocaloriginofdomesticpigsintheupstreamregionoftheyang
MitochondrialDNAevidenceindicatesthelocaloriginofdomesticpigsintheupstreamregionoftheyang
MitochondrialDNAevidenceindicatesthelocaloriginofdomesticpigsintheupstreamregionoftheyang
MitochondrialDNAevidenceindicatesthelocaloriginofdomesticpigsintheupstreamregionoftheyang
MitochondrialDNAevidenceindicatesthelocaloriginofdomesticpigsintheupstreamregionoftheyang
MitochondrialDNAevidenceindicatesthelocaloriginofdomesticpigsintheupstreamregionoftheyang
MitochondrialDNAevidenceindicatesthelocaloriginofdomesticpigsintheupstreamregionoftheyang
MitochondrialDNAevidenceindicatesthelocaloriginofdomesticpigsintheupstreamregionoftheyang
MitochondrialDNAevidenceindicatesthelocaloriginofdomesticpigsintheupstreamregionoftheyang
MitochondrialDNAevidenceindicatesthelocaloriginofdomesticpigsintheupstreamregionoftheyang
MitochondrialDNAevidenceindicatesthelocaloriginofdomesticpigsintheupstreamregionoftheyang
MitochondrialDNAevidenceindicatesthelocaloriginofdomesticpigsintheupstreamregionoftheyang
MitochondrialDNAevidenceindicatesthelocaloriginofdomesticpigsintheupstreamregionoftheyang
MitochondrialDNAevidenceindicatesthelocaloriginofdomesticpigsintheupstreamregionoftheyang
MitochondrialDNAevidenceindicatesthelocaloriginofdomesticpigsintheupstreamregionoftheyang
MitochondrialDNAevidenceindicatesthelocaloriginofdomesticpigsintheupstreamregionoftheyang
MitochondrialDNAevidenceindicatesthelocaloriginofdomesticpigsintheupstreamregionoftheyang
MitochondrialDNAevidenceindicatesthelocaloriginofdomesticpigsintheupstreamregionoftheyang
MitochondrialDNAevidenceindicatesthelocaloriginofdomesticpigsintheupstreamregionoftheyang
MitochondrialDNAevidenceindicatesthelocaloriginofdomesticpigsintheupstreamregionoftheyang
MitochondrialDNAevidenceindicatesthelocaloriginofdomesticpigsintheupstreamregionoftheyang
MitochondrialDNAevidenceindicatesthelocaloriginofdomesticpigsintheupstreamregionoftheyang
MitochondrialDNAevidenceindicatesthelocaloriginofdomesticpigsintheupstreamregionoftheyang
MitochondrialDNAevidenceindicatesthelocaloriginofdomesticpigsintheupstreamregionoftheyang
MitochondrialDNAevidenceindicatesthelocaloriginofdomesticpigsintheupstreamregionoftheyang
MitochondrialDNAevidenceindicatesthelocaloriginofdomesticpigsintheupstreamregionoftheyang
MitochondrialDNAevidenceindicatesthelocaloriginofdomesticpigsintheupstreamregionoftheyang
MitochondrialDNAevidenceindicatesthelocaloriginofdomesticpigsintheupstreamregionoftheyang
PopulationphylogenomicanalysisofmitochondrialDNAinwildboarsanddomesticpigsrevealedmultiple
PopulationphylogenomicanalysisofmitochondrialDNAinwildboarsanddomesticpigsrevealedmultiple
PopulationphylogenomicanalysisofmitochondrialDNAinwildboarsanddomesticpigsrevealedmultiple
PopulationphylogenomicanalysisofmitochondrialDNAinwildboarsanddomesticpigsrevealedmultiple
Phylogenetic analyses of Chinese domestic pigs
PhylogeneticrelationshipsofAsianandEuropeanpigbreedsdeterminedbymitochondrialDNAD-loopseq
MitochondrialdiversityinEuropeanandChinesepigsisconsistentwithpopulationexpansionsthatocc
MitochondrialdiversityinEuropeanandChinesepigsisconsistentwithpopulationexpansionsthatocc
MitochondrialdiversityinEuropeanandChinesepigsisconsistentwithpopulationexpansionsthatocc
MitochondrialdiversityinEuropeanandChinesepigsisconsistentwithpopulationexpansionsthatocc
MitochondrialdiversityinEuropeanandChinesepigsisconsistentwithpopulationexpansionsthatocc
MitochondrialDNAevidenceindicatesthelocaloriginofdomesticpigsintheupstreamregionoftheyang
MitochondrialDNAevidenceindicatesthelocaloriginofdomesticpigsintheupstreamregionoftheyang
MitochondrialDNAevidenceindicatesthelocaloriginofdomesticpigsintheupstreamregionoftheyang
MitochondrialDNAevidenceindicatesthelocaloriginofdomesticpigsintheupstreamregionoftheyang
MitochondrialDNAevidenceindicatesthelocaloriginofdomesticpigsintheupstreamregionoftheyang
MitochondrialDNAevidenceindicatesthelocaloriginofdomesticpigsintheupstreamregionoftheyang
MitochondrialDNAevidenceindicatesthelocaloriginofdomesticpigsintheupstreamregionoftheyang
MitochondrialDNAevidenceindicatesthelocaloriginofdomesticpigsintheupstreamregionoftheyang
MitochondrialDNAevidenceindicatesthelocaloriginofdomesticpigsintheupstreamregionoftheyang

MitochondrialDNAevidenceindicatesthelocaloriginofdomesticpigsintheupstreamregionoftheyang[illegible]
MitochondrialDNAevidenceindicatesthelocaloriginofdomesticpigsintheupstreamregionoftheyang[illegible]
MitochondrialDNAevidenceindicatesthelocaloriginofdomesticpigsintheupstreamregionoftheyang[illegible]
MitochondrialDNAevidenceindicatesthelocaloriginofdomesticpigsintheupstreamregionoftheyang[illegible]
MitochondrialDNAevidenceindicatesthelocaloriginofdomesticpigsintheupstreamregionoftheyang[illegible]
MitochondrialDNAevidenceindicatesthelocaloriginofdomesticpigsintheupstreamregionoftheyang[illegible]
MitochondrialDNAevidenceindicatesthelocaloriginofdomesticpigsintheupstreamregionoftheyang[illegible]
MitochondrialDNAevidenceindicatesthelocaloriginofdomesticpigsintheupstreamregionoftheyang[illegible]
MitochondrialDNAevidenceindicatesthelocaloriginofdomesticpigsintheupstreamregionoftheyang[illegible]
MitochondrialDNAevidenceindicatesthelocaloriginofdomesticpigsintheupstreamregionoftheyang[illegible]
MitochondrialDNAevidenceindicatesthelocaloriginofdomesticpigsintheupstreamregionoftheyang[illegible]
MitochondrialDNAevidenceindicatesthelocaloriginofdomesticpigsintheupstreamregionoftheyang[illegible]
MitochondrialDNAevidenceindicatesthelocaloriginofdomesticpigsintheupstreamregionoftheyang[illegible]
MitochondrialDNAevidenceindicatesthelocaloriginofdomesticpigsintheupstreamregionoftheyang[illegible]
MitochondrialDNAevidenceindicatesthelocaloriginofdomesticpigsintheupstreamregionoftheyang[illegible]
MitochondrialDNAevidenceindicatesthelocaloriginofdomesticpigsintheupstreamregionoftheyang[illegible]
MitochondrialDNAevidenceindicatesthelocaloriginofdomesticpigsintheupstreamregionoftheyang[illegible]
MitochondrialDNAevidenceindicatesthelocaloriginofdomesticpigsintheupstreamregionoftheyang[illegible]
MitochondrialDNAevidenceindicatesthelocaloriginofdomesticpigsintheupstreamregionoftheyang[illegible]
MitochondrialDNAevidenceindicatesthelocaloriginofdomesticpigsintheupstreamregionoftheyang[illegible]
MitochondrialDNAevidenceindicatesthelocaloriginofdomesticpigsintheupstreamregionoftheyang[illegible]
MitochondrialDNAevidenceindicatesthelocaloriginofdomesticpigsintheupstreamregionoftheyang[illegible]
MitochondrialDNAevidenceindicatesthelocaloriginofdomesticpigsintheupstreamregionoftheyang[illegible]
MitochondrialDNAevidenceindicatesthelocaloriginofdomesticpigsintheupstreamregionoftheyang[illegible]
MitochondrialDNAevidenceindicatesthelocaloriginofdomesticpigsintheupstreamregionoftheyang[illegible]
MitochondrialDNAevidenceindicatesthelocaloriginofdomesticpigsintheupstreamregionoftheyang[illegible]
MitochondrialDNAevidenceindicatesthelocaloriginofdomesticpigsintheupstreamregionoftheyang[illegible]
MitochondrialDNAevidenceindicatesthelocaloriginofdomesticpigsintheupstreamregionoftheyang[illegible]
MitochondrialDNAevidenceindicatesthelocaloriginofdomesticpigsintheupstreamregionoftheyang[illegible]
MitochondrialDNAevidenceindicatesthelocaloriginofdomesticpigsintheupstreamregionoftheyang[illegible]
MitochondrialDNAevidenceindicatesthelocaloriginofdomesticpigsintheupstreamregionoftheyang[illegible]
MitochondrialDNAevidenceindicatesthelocaloriginofdomesticpigsintheupstreamregionoftheyang[illegible]
MitochondrialDNAevidenceindicatesthelocaloriginofdomesticpigsintheupstreamregionoftheyang[illegible]
MitochondrialDNAevidenceindicatesthelocaloriginofdomesticpigsintheupstreamregionoftheyang[illegible]
MitochondrialDNAevidenceindicatesthelocaloriginofdomesticpigsintheupstreamregionoftheyang[illegible]
MitochondrialDNAevidenceindicatesthelocaloriginofdomesticpigsintheupstreamregionoftheyang[illegible]
MitochondrialDNAevidenceindicatesthelocaloriginofdomesticpigsintheupstreamregionoftheyang[illegible]
MitochondrialDNAevidenceindicatesthelocaloriginofdomesticpigsintheupstreamregionoftheyang[illegible]
MitochondrialDNAevidenceindicatesthelocaloriginofdomesticpigsintheupstreamregionoftheyang[illegible]
MitochondrialDNAevidenceindicatesthelocaloriginofdomesticpigsintheupstreamregionoftheyang[illegible]
MitochondrialDNAevidenceindicatesthelocaloriginofdomesticpigsintheupstreamregionoftheyang[illegible]
MitochondrialDNAevidenceindicatesthelocaloriginofdomesticpigsintheupstreamregionoftheyang[illegible]
MitochondrialDNAevidenceindicatesthelocaloriginofdomesticpigsintheupstreamregionoftheyang[illegible]
MitochondrialDNAevidenceindicatesthelocaloriginofdomesticpigsintheupstreamregionoftheyang[illegible]
MitochondrialDNAevidenceindicatesthelocaloriginofdomesticpigsintheupstreamregionoftheyang[illegible]
MitochondrialDNAevidenceindicatesthelocaloriginofdomesticpigsintheupstreamregionoftheyang[illegible]
MitochondrialDNAevidenceindicatesthelocaloriginofdomesticpigsintheupstreamregionoftheyang[illegible]
MitochondrialDNAevidenceindicatesthelocaloriginofdomesticpigsintheupstreamregionoftheyang[illegible]
MitochondrialDNAevidenceindicatesthelocaloriginofdomesticpigsintheupstreamregionoftheyang[illegible]
MitochondrialDNAevidenceindicatesthelocaloriginofdomesticpigsintheupstreamregionoftheyang[illegible]
MitochondrialDNAevidenceindicatesthelocaloriginofdomesticpigsintheupstreamregionoftheyang[illegible]
MitochondrialDNAevidenceindicatesthelocaloriginofdomesticpigsintheupstreamregionoftheyang[illegible]
Populationphylogenomicanalysisofmitochondrial DNAinwildboarsanddomesticpigsrevealedmultipl[illegible]
Populationphylogenomicanalysisofmitochondrial DNAinwildboarsanddomesticpigsrevealedmultipl[illegible]
Populationphylogenomicanalysisofmitochondrial DNAinwildboarsanddomesticpigsrevealedmultipl[illegible]
Populationphylogenomicanalysisofmitochondrial DNAinwildboarsanddomesticpigsrevealedmultipl[illegible]
Populationphylogenomicanalysisofmitochondrial DNAinwildboarsanddomesticpigsrevealedmultipl[illegible]
Populationphylogenomicanalysisofmitochondrial DNAinwildboarsanddomesticpigsrevealedmultipl[illegible]
Populationphylogenomicanalysisofmitochondrial DNAinwildboarsanddomesticpigsrevealedmultipl[illegible]
Populationphylogenomicanalysisofmitochondrial DNAinwildboarsanddomesticpigsrevealedmultipl[illegible]
Populationphylogenomicanalysisofmitochondrial DNAinwildboarsanddomesticpigsrevealedmultipl[illegible]
Populationphylogenomicanalysisofmitochondrial DNAinwildboarsanddomesticpigsrevealedmultipl[illegible]
Populationphylogenomicanalysisofmitochondrial DNAinwildboarsanddomesticpigsrevealedmultipl[illegible]
Populationphylogenomicanalysisofmitochondrial DNAinwildboarsanddomesticpigsrevealedmultipl[illegible]
Populationphylogenomicanalysisofmitochondrial DNAinwildboarsanddomesticpigsrevealedmultipl[illegible]
Populationphylogenomicanalysisofmitochondrial DNAinwildboarsanddomesticpigsrevealedmultipl[illegible]
Populationphylogenomicanalysisofmitochondrial DNAinwildboarsanddomesticpigsrevealedmultipl[illegible]
CharacterizationoftheEuropeantypeofmaternallineageevidentinextantJejunativepigsJOURNALGen[illegible]
MitochondrialDNAevidenceindicatesthelocaloriginofdomesticpigsintheupstreamregionoftheyang[illegible]
MitochondrialDNAevidenceindicatesthelocaloriginofdomesticpigsintheupstreamregionoftheyang[illegible]

MitochondrialDNAevidenceindicatesthelocaloriginofdomesticpigsintheupstreamregionoftheyang
MitochondrialDNAevidenceindicatesthelocaloriginofdomesticpigsintheupstreamregionoftheyang
MitochondrialDNAevidenceindicatesthelocaloriginofdomesticpigsintheupstreamregionoftheyang
MitochondrialDNAevidenceindicatesthelocaloriginofdomesticpigsintheupstreamregionoftheyang
MitochondrialDNAevidenceindicatesthelocaloriginofdomesticpigsintheupstreamregionoftheyang
MitochondrialDNAevidenceindicatesthelocaloriginofdomesticpigsintheupstreamregionoftheyang
MitochondrialDNAevidenceindicatesthelocaloriginofdomesticpigsintheupstreamregionoftheyang
MitochondrialDNAevidenceindicatesthelocaloriginofdomesticpigsintheupstreamregionoftheyang
MitochondrialDNAevidenceindicatesthelocaloriginofdomesticpigsintheupstreamregionoftheyang
MitochondrialDNAevidenceindicatesthelocaloriginofdomesticpigsintheupstreamregionoftheyang
MitochondrialDNAevidenceindicatesthelocaloriginofdomesticpigsintheupstreamregionoftheyang
MitochondrialDNAevidenceindicatesthelocaloriginofdomesticpigsintheupstreamregionoftheyang
MitochondrialDNAevidenceindicatesthelocaloriginofdomesticpigsintheupstreamregionoftheyang
MitochondrialDNAevidenceindicatesthelocaloriginofdomesticpigsintheupstreamregionoftheyang
MitochondrialDNAevidenceindicatesthelocaloriginofdomesticpigsintheupstreamregionoftheyang
MitochondrialDNAevidenceindicatesthelocaloriginofdomesticpigsintheupstreamregionoftheyang
MitochondrialDNAevidenceindicatesthelocaloriginofdomesticpigsintheupstreamregionoftheyang
MitochondrialDNAevidenceindicatesthelocaloriginofdomesticpigsintheupstreamregionoftheyang
MitochondrialDNAevidenceindicatesthelocaloriginofdomesticpigsintheupstreamregionoftheyang
MitochondrialDNAevidenceindicatesthelocaloriginofdomesticpigsintheupstreamregionoftheyang
MitochondrialDNAevidenceindicatesthelocaloriginofdomesticpigsintheupstreamregionoftheyang
MitochondrialDNAevidenceindicatesthelocaloriginofdomesticpigsintheupstreamregionoftheyang
MitochondrialDNAevidenceindicatesthelocaloriginofdomesticpigsintheupstreamregionoftheyang
MitochondrialDNAevidenceindicatesthelocaloriginofdomesticpigsintheupstreamregionoftheyang
MitochondrialDNAevidenceindicatesthelocaloriginofdomesticpigsintheupstreamregionoftheyang
MitochondrialDNAevidenceindicatesthelocaloriginofdomesticpigsintheupstreamregionoftheyang
MitochondrialDNAevidenceindicatesthelocaloriginofdomesticpigsintheupstreamregionoftheyang
MitochondrialDNAevidenceindicatesthelocaloriginofdomesticpigsintheupstreamregionoftheyang
MitochondrialDNAevidenceindicatesthelocaloriginofdomesticpigsintheupstreamregionoftheyang
MitochondrialDNAevidenceindicatesthelocaloriginofdomesticpigsintheupstreamregionoftheyang
MitochondrialDNAevidenceindicatesthelocaloriginofdomesticpigsintheupstreamregionoftheyang
MitochondrialDNAevidenceindicatesthelocaloriginofdomesticpigsintheupstreamregionoftheyang
MitochondrialDNAevidenceindicatesthelocaloriginofdomesticpigsintheupstreamregionoftheyang
MitochondrialDNAevidenceindicatesthelocaloriginofdomesticpigsintheupstreamregionoftheyang
MitochondrialDNAevidenceindicatesthelocaloriginofdomesticpigsintheupstreamregionoftheyang
MitochondrialDNAevidenceindicatesthelocaloriginofdomesticpigsintheupstreamregionoftheyang
MitochondrialDNAevidenceindicatesthelocaloriginofdomesticpigsintheupstreamregionoftheyang
MitochondrialDNAevidenceindicatesthelocaloriginofdomesticpigsintheupstreamregionoftheyang
MitochondrialDNAevidenceindicatesthelocaloriginofdomesticpigsintheupstreamregionoftheyang
MitochondrialDNAevidenceindicatesthelocaloriginofdomesticpigsintheupstreamregionoftheyang
MitochondrialDNAevidenceindicatesthelocaloriginofdomesticpigsintheupstreamregionoftheyang
MitochondrialDNAevidenceindicatesthelocaloriginofdomesticpigsintheupstreamregionoftheyang
MitochondrialDNAevidenceindicatesthelocaloriginofdomesticpigsintheupstreamregionoftheyang
MitochondrialDNAevidenceindicatesthelocaloriginofdomesticpigsintheupstreamregionoftheyang
MitochondrialDNAevidenceindicatesthelocaloriginofdomesticpigsintheupstreamregionoftheyang
MitochondrialDNAevidenceindicatesthelocaloriginofdomesticpigsintheupstreamregionoftheyang
MitochondrialDNAevidenceindicatesthelocaloriginofdomesticpigsintheupstreamregionoftheyang
MitochondrialDNAevidenceindicatesthelocaloriginofdomesticpigsintheupstreamregionoftheyang
PopulationphylogenomicanalysisofmitochondrialDNAinwildboarsanddomesticpigsrevealedmultiple
PopulationphylogenomicanalysisofmitochondrialDNAinwildboarsanddomesticpigsrevealedmultiple
PopulationphylogenomicanalysisofmitochondrialDNAinwildboarsanddomesticpigsrevealedmultiple
PopulationphylogenomicanalysisofmitochondrialDNAinwildboarsanddomesticpigsrevealedmultiple
PopulationphylogenomicanalysisofmitochondrialDNAinwildboarsanddomesticpigsrevealedmultiple
PopulationphylogenomicanalysisofmitochondrialDNAinwildboarsanddomesticpigsrevealedmultiple
PopulationphylogenomicanalysisofmitochondrialDNAinwildboarsanddomesticpigsrevealedmultiple
PopulationphylogenomicanalysisofmitochondrialDNAinwildboarsanddomesticpigsrevealedmultiple
PopulationphylogenomicanalysisofmitochondrialDNAinwildboarsanddomesticpigsrevealedmultiple
PopulationphylogenomicanalysisofmitochondrialDNAinwildboarsanddomesticpigsrevealedmultiple
CharacterizationoftheEuropeantypeofmaternallineageevidentinextantJejunativepigsJOURNALGene

PopulationphylogenomicanalysisofmitochondrialDNAinwildboarsanddomesticpigsrevealedmultipl
PopulationphylogenomicanalysisofmitochondrialDNAinwildboarsanddomesticpigsrevealedmultipl
PopulationphylogenomicanalysisofmitochondrialDNAinwildboarsanddomesticpigsrevealedmultipl
PopulationphylogenomicanalysisofmitochondrialDNAinwildboarsanddomesticpigsrevealedmultipl
PopulationphylogenomicanalysisofmitochondrialDNAinwildboarsanddomesticpigsrevealedmultipl
PopulationphylogenomicanalysisofmitochondrialDNAinwildboarsanddomesticpigsrevealedmultipl
MitochondrialDNAevidenceindicatesthelocaloriginofdomesticpigsintheupstreamregionoftheyang
MitochondrialDNAevidenceindicatesthelocaloriginofdomesticpigsintheupstreamregionoftheyang
MitochondrialDNAevidenceindicatesthelocaloriginofdomesticpigsintheupstreamregionoftheyang
MitochondrialDNAevidenceindicatesthelocaloriginofdomesticpigsintheupstreamregionoftheyang
MitochondrialDNAevidenceindicatesthelocaloriginofdomesticpigsintheupstreamregionoftheyang
MitochondrialDNAevidenceindicatesthelocaloriginofdomesticpigsintheupstreamregionoftheyang
MitochondrialDNAevidenceindicatesthelocaloriginofdomesticpigsintheupstreamregionoftheyang
MitochondrialDNAevidenceindicatesthelocaloriginofdomesticpigsintheupstreamregionoftheyang
MitochondrialDNAevidenceindicatesthelocaloriginofdomesticpigsintheupstreamregionoftheyang
MitochondrialDNAevidenceindicatesthelocaloriginofdomesticpigsintheupstreamregionoftheyang
MitochondrialDNAevidenceindicatesthelocaloriginofdomesticpigsintheupstreamregionoftheyang
MitochondrialDNAevidenceindicatesthelocaloriginofdomesticpigsintheupstreamregionoftheyang
MitochondrialDNAevidenceindicatesthelocaloriginofdomesticpigsintheupstreamregionoftheyang
MitochondrialDNAevidenceindicatesthelocaloriginofdomesticpigsintheupstreamregionoftheyang
MitochondrialDNAevidenceindicatesthelocaloriginofdomesticpigsintheupstreamregionoftheyang
MitochondrialDNAevidenceindicatesthelocaloriginofdomesticpigsintheupstreamregionoftheyang
MitochondrialDNAevidenceindicatesthelocaloriginofdomesticpigsintheupstreamregionoftheyang
MitochondrialDNAevidenceindicatesthelocaloriginofdomesticpigsintheupstreamregionoftheyang
MitochondrialDNAevidenceindicatesthelocaloriginofdomesticpigsintheupstreamregionoftheyang
MitochondrialDNAevidenceindicatesthelocaloriginofdomesticpigsintheupstreamregionoftheyang
MitochondrialDNAevidenceindicatesthelocaloriginofdomesticpigsintheupstreamregionoftheyang
MitochondrialDNAevidenceindicatesthelocaloriginofdomesticpigsintheupstreamregionoftheyang
MitochondrialDNAevidenceindicatesthelocaloriginofdomesticpigsintheupstreamregionoftheyang
MitochondrialDNAevidenceindicatesthelocaloriginofdomesticpigsintheupstreamregionoftheyang
MitochondrialDNAevidenceindicatesthelocaloriginofdomesticpigsintheupstreamregionoftheyang
MitochondrialDNAevidenceindicatesthelocaloriginofdomesticpigsintheupstreamregionoftheyang
MitochondrialDNAevidenceindicatesthelocaloriginofdomesticpigsintheupstreamregionoftheyang
MitochondrialDNAevidenceindicatesthelocaloriginofdomesticpigsintheupstreamregionoftheyang
MitochondrialDNAevidenceindicatesthelocaloriginofdomesticpigsintheupstreamregionoftheyang
MitochondrialDNAevidenceindicatesthelocaloriginofdomesticpigsintheupstreamregionoftheyang
MitochondrialDNAevidenceindicatesthelocaloriginofdomesticpigsintheupstreamregionoftheyang
MitochondrialDNAevidenceindicatesthelocaloriginofdomesticpigsintheupstreamregionoftheyang
MitochondrialDNAevidenceindicatesthelocaloriginofdomesticpigsintheupstreamregionoftheyang
MitochondrialDNAevidenceindicatesthelocaloriginofdomesticpigsintheupstreamregionoftheyang
MitochondrialDNAevidenceindicatesthelocaloriginofdomesticpigsintheupstreamregionoftheyang
MitochondrialDNAevidenceindicatesthelocaloriginofdomesticpigsintheupstreamregionoftheyang
MitochondrialDNAevidenceindicatesthelocaloriginofdomesticpigsintheupstreamregionoftheyang
PhylogeneticanalysesofChinesedomesticpigs

esGenomics33(2),111-117(2011)
urredpriortodomesticationJOURNALProc.Biol.Sci.273(1595),1803-1810(2006)
urredpriortodomesticationJOURNALProc.Biol.Sci.273(1595),1803-1810(2006)
urredpriortodomesticationJOURNALProc.Biol.Sci.273(1595),1803-1810(2006)
urredpriortodomesticationJOURNALProc.Biol.Sci.273(1595),1803-1810(2006)
urredpriortodomesticationJOURNALProc.Biol.Sci.273(1595),1803-1810(2006)
urredpriortodomesticationJOURNALProc.Biol.Sci.273(1595),1803-1810(2006)
urredpriortodomesticationJOURNALProc.Biol.Sci.273(1595),1803-1810(2006)
urredpriortodomesticationJOURNALProc.Biol.Sci.273(1595),1803-1810(2006)
urredpriortodomesticationJOURNALProc.Biol.Sci.273(1595),1803-1810(2006)
urredpriortodomesticationJOURNALProc.Biol.Sci.273(1595),1803-1810(2006)
edomesticationeventsinEastAsiaJOURNALGenomeBiol.8(11),R245(2007)
edomesticationeventsinEastAsiaJOURNALGenomeBiol.8(11),R245(2007)
edomesticationeventsinEastAsiaJOURNALGenomeBiol.8(11),R245(2007)
edomesticationeventsinEastAsiaGenomeBiol.8(11),R245(2007)
edomesticationeventsinEastAsiaGenomeBiol.8(11),R245(2007)
edomesticationeventsinEastAsiaGenomeBiol.8(11),R245(2007)
edomesticationeventsinEastAsiaGenomeBiol.8(11),R245(2007)
edomesticationeventsinEastAsiaGenomeBiol.8(11),R245(2007)
edomesticationeventsinEastAsiaGenomeBiol.8(11),R245(2007)
edomesticationeventsinEastAsiaGenomeBiol.8(11),R245(2007)
edomesticationeventsinEastAsiaGenomeBiol.8(11),R245(2007)
edomesticationeventsinEastAsiaGenomeBiol.8(11),R245(2007)
edomesticationeventsinEastAsiaGenomeBiol.8(11),R245(2007)
edomesticationeventsinEastAsiaGenomeBiol.8(11),R245(2007)
edomesticationeventsinEastAsiaGenomeBiol.8(11),R245(2007)
edomesticationeventsinEastAsiaGenomeBiol.8(11),R245(2007)
edomesticationeventsinEastAsiaGenomeBiol.8(11),R245(2007)
edomesticationeventsinEastAsiaGenomeBiol.8(11),R245(2007)
edomesticationeventsinEastAsiaGenomeBiol.8(11),R245(2007)
edomesticationeventsinEastAsiaGenomeBiol.8(11),R245(2007)
edomesticationeventsinEastAsiaGenomeBiol.8(11),R245(2007)
edomesticationeventsinEastAsiaGenomeBiol.8(11),R245(2007)
edomesticationeventsinEastAsiaGenomeBiol.8(11),R245(2007)
edomesticationeventsinEastAsiaGenomeBiol.8(11),R245(2007)
edomesticationeventsinEastAsiaGenomeBiol.8(11),R245(2007)
edomesticationeventsinEastAsiaGenomeBiol.8(11),R245(2007)
edomesticationeventsinEastAsiaGenomeBiol.8(11),R245(2007)
edomesticationeventsinEastAsiaGenomeBiol.8(11),R245(2007)
edomesticationeventsinEastAsiaGenomeBiol.8(11),R245(2007)

edomesticationeventsinEastAsiaGenomeBiol.8(11),R245(2007)
edomesticationeventsinEastAsiaGenomeBiol.8(11),R245(2007)
edomesticationeventsinEastAsiaGenomeBiol.8(11),R245(2007)
edomesticationeventsinEastAsiaGenomeBiol.8(11),R245(2007)
edomesticationeventsinEastAsiaGenomeBiol.8(11),R245(2007)
edomesticationeventsinEastAsiaGenomeBiol.8(11),R245(2007)
edomesticationeventsinEastAsiaGenomeBiol.8(11),R245(2007)
edomesticationeventsinEastAsiaGenomeBiol.8(11),R245(2007)
edomesticationeventsinEastAsiaGenomeBiol.8(11),R245(2007)
edomesticationeventsinEastAsiaGenomeBiol.8(11),R245(2007)
edomesticationeventsinEastAsiaGenomeBiol.8(11),R245(2007)
edomesticationeventsinEastAsiaGenomeBiol.8(11),R245(2007)
edomesticationeventsinEastAsiaGenomeBiol.8(11),R245(2007)
edomesticationeventsinEastAsiaGenomeBiol.8(11),R245(2007)
edomesticationeventsinEastAsiaGenomeBiol.8(11),R245(2007)
edomesticationeventsinEastAsiaGenomeBiol.8(11),R245(2007)
edomesticationeventsinEastAsiaGenomeBiol.8(11),R245(2007)
edomesticationeventsinEastAsiaGenomeBiol.8(11),R245(2007)
edomesticationeventsinEastAsiaGenomeBiol.8(11),R245(2007)
(12),E28215(2011)
(12),E28215(2011)
(12),E28215(2011)
(12),E28215(2011)
(12),E28215(2011)
(12),E28215(2011)
(12),E28215(2011)
(12),E28215(2011)
(12),E28215(2011)
(12),E28215(2011)
(12),E28215(2011)
(12),E28215(2011)
(12),E28215(2011)
(12),E28215(2011)
(12),E28215(2011)
(12),E28215(2011)
(12),E28215(2011)
(12),E28215(2011)
(12),E28215(2011)
edomesticationeventsinEastAsiaGenomeBiol.8(11),R245(2007)
edomesticationeventsinEastAsiaGenomeBiol.8(11),R245(2007)
edomesticationeventsinEastAsiaGenomeBiol.8(11),R245(2007)
edomesticationeventsinEastAsiaGenomeBiol.8(11),R245(2007)
edomesticationeventsinEastAsiaGenomeBiol.8(11),R245(2007)
edomesticationeventsinEastAsiaGenomeBiol.8(11),R245(2007)
edomesticationeventsinEastAsiaGenomeBiol.8(11),R245(2007)
edomesticationeventsinEastAsiaGenomeBiol.8(11),R245(2007)
edomesticationeventsinEastAsiaGenomeBiol.8(11),R245(2007)
edomesticationeventsinEastAsiaGenomeBiol.8(11),R245(2007)
edomesticationeventsinEastAsiaGenomeBiol.8(11),R245(2007)
edomesticationeventsinEastAsiaGenomeBiol.8(11),R245(2007)
edomesticationeventsinEastAsiaGenomeBiol.8(11),R245(2007)
edomesticationeventsinEastAsiaGenomeBiol.8(11),R245(2007)
edomesticationeventsinEastAsiaGenomeBiol.8(11),R245(2007)

edomesticationeventsinEastAsiaGenomeBiol.8(11),R245(2007)
(12),E28215(2011)
(12),E28215(2011)
(12),E28215(2011)
(12),E28215(2011)
(12),E28215(2011)
(12),E28215(2011)
(12),E28215(2011)
(12),E28215(2011)
(12),E28215(2011)
(12),E28215(2011)

edomesticationeventsinEastAsiaGenomeBiol.8(11),R245(2007)
edomesticationeventsinEastAsiaGenomeBiol.8(11),R245(2007)
edomesticationeventsinEastAsiaGenomeBiol.8(11),R245(2007)
edomesticationeventsinEastAsiaGenomeBiol.8(11),R245(2007)
edomesticationeventsinEastAsiaGenomeBiol.8(11),R245(2007)
edomesticationeventsinEastAsiaGenomeBiol.8(11),R245(2007)
edomesticationeventsinEastAsiaGenomeBiol.8(11),R245(2007)
edomesticationeventsinEastAsiaGenomeBiol.8(11),R245(2007)
edomesticationeventsinEastAsiaGenomeBiol.8(11),R245(2007)
edomesticationeventsinEastAsiaGenomeBiol.8(11),R245(2007)
edomesticationeventsinEastAsiaGenomeBiol.8(11),R245(2007)
edomesticationeventsinEastAsiaGenomeBiol.8(11),R245(2007)
edomesticationeventsinEastAsiaGenomeBiol.8(11),R245(2007)
edomesticationeventsinEastAsiaGenomeBiol.8(11),R245(2007)
edomesticationeventsinEastAsiaGenomeBiol.8(11),R245(2007)
edomesticationeventsinEastAsiaGenomeBiol.8(11),R245(2007)
edomesticationeventsinEastAsiaGenomeBiol.8(11),R245(2007)
edomesticationeventsinEastAsiaGenomeBiol.8(11),R245(2007)
edomesticationeventsinEastAsiaGenomeBiol.8(11),R245(2007)
edomesticationeventsinEastAsiaGenomeBiol.8(11),R245(2007)
edomesticationeventsinEastAsiaGenomeBiol.8(11),R245(2007)
edomesticationeventsinEastAsiaGenomeBiol.8(11),R245(2007)
edomesticationeventsinEastAsiaGenomeBiol.8(11),R245(2007)
edomesticationeventsinEastAsiaGenomeBiol.8(11),R245(2007)
edomesticationeventsinEastAsiaGenomeBiol.8(11),R245(2007)
edomesticationeventsinEastAsiaGenomeBiol.8(11),R245(2007)
edomesticationeventsinEastAsiaGenomeBiol.8(11),R245(2007)
edomesticationeventsinEastAsiaGenomeBiol.8(11),R245(2007)
edomesticationeventsinEastAsiaGenomeBiol.8(11),R245(2007)
edomesticationeventsinEastAsiaGenomeBiol.8(11),R245(2007)
edomesticationeventsinEastAsiaGenomeBiol.8(11),R245(2007)
edomesticationeventsinEastAsiaGenomeBiol.8(11),R245(2007)
edomesticationeventsinEastAsiaGenomeBiol.8(11),R245(2007)
edomesticationeventsinEastAsiaGenomeBiol.8(11),R245(2007)
edomesticationeventsinEastAsiaGenomeBiol.8(11),R245(2007)
edomesticationeventsinEastAsiaGenomeBiol.8(11),R245(2007)
edomesticationeventsinEastAsiaGenomeBiol.8(11),R245(2007)
edomesticationeventsinEastAsiaGenomeBiol.8(11),R245(2007)
edomesticationeventsinEastAsiaGenomeBiol.8(11),R245(2007)
edomesticationeventsinEastAsiaGenomeBiol.8(11),R245(2007)
(12),E28215(2011)
(12),E28215(2011)
(12),E28215(2011)
(12),E28215(2011)
(12),E28215(2011)
(12),E28215(2011)

(12),E28215(2011)
(12),E28215(2011)
(12),E28215(2011)
(12),E28215(2011)
(12),E28215(2011)
(12),E28215(2011)
(12),E28215(2011)
(12),E28215(2011)
(12),E28215(2011)
(12),E28215(2011)
(12),E28215(2011)
(12),E28215(2011)
(12),E28215(2011)
(12),E28215(2011)
(12),E28215(2011)
(12),E28215(2011)
(12),E28215(2011)
(12),E28215(2011)
(12),E28215(2011)
(12),E28215(2011)
edomesticationeventsinEastAsiaJOURNALGenomeBiol.8(11),R245(2007)
edomesticationeventsinEastAsiaJOURNALGenomeBiol.8(11),R245(2007)
edomesticationeventsinEastAsiaGenomeBiol.8(11),R245(2007)
edomesticationeventsinEastAsiaGenomeBiol.8(11),R245(2007)
edomesticationeventsinEastAsiaGenomeBiol.8(11),R245(2007)
edomesticationeventsinEastAsiaGenomeBiol.8(11),R245(2007)
edomesticationeventsinEastAsiaGenomeBiol.8(11),R245(2007)
edomesticationeventsinEastAsiaGenomeBiol.8(11),R245(2007)
edomesticationeventsinEastAsiaGenomeBiol.8(11),R245(2007)
edomesticationeventsinEastAsiaGenomeBiol.8(11),R245(2007)
edomesticationeventsinEastAsiaGenomeBiol.8(11),R245(2007)
edomesticationeventsinEastAsiaGenomeBiol.8(11),R245(2007)
edomesticationeventsinEastAsiaGenomeBiol.8(11),R245(2007)
edomesticationeventsinEastAsiaGenomeBiol.8(11),R245(2007)
edomesticationeventsinEastAsiaGenomeBiol.8(11),R245(2007)
edomesticationeventsinEastAsiaGenomeBiol.8(11),R245(2007)
edomesticationeventsinEastAsiaGenomeBiol.8(11),R245(2007)
edomesticationeventsinEastAsiaGenomeBiol.8(11),R245(2007)
edomesticationeventsinEastAsiaGenomeBiol.8(11),R245(2007)
edomesticationeventsinEastAsiaGenomeBiol.8(11),R245(2007)
edomesticationeventsinEastAsiaGenomeBiol.8(11),R245(2007)
edomesticationeventsinEastAsiaGenomeBiol.8(11),R245(2007)
edomesticationeventsinEastAsiaGenomeBiol.8(11),R245(2007)
edomesticationeventsinEastAsiaGenomeBiol.8(11),R245(2007)
edomesticationeventsinEastAsiaGenomeBiol.8(11),R245(2007)
edomesticationeventsinEastAsiaGenomeBiol.8(11),R245(2007)
edomesticationeventsinEastAsiaGenomeBiol.8(11),R245(2007)
edomesticationeventsinEastAsiaGenomeBiol.8(11),R245(2007)
edomesticationeventsinEastAsiaGenomeBiol.8(11),R245(2007)
edomesticationeventsinEastAsiaGenomeBiol.8(11),R245(2007)
edomesticationeventsinEastAsiaGenomeBiol.8(11),R245(2007)
edomesticationeventsinEastAsiaGenomeBiol.8(11),R245(2007)
edomesticationeventsinEastAsiaGenomeBiol.8(11),R245(2007)
edomesticationeventsinEastAsiaGenomeBiol.8(11),R245(2007)
edomesticationeventsinEastAsiaGenomeBiol.8(11),R245(2007)
edomesticationeventsinEastAsiaGenomeBiol.8(11),R245(2007)
edomesticationeventsinEastAsiaGenomeBiol.8(11),R245(2007)
edomesticationeventsinEastAsiaGenomeBiol.8(11),R245(2007)
edomesticationeventsinEastAsiaGenomeBiol.8(11),R245(2007)
edomesticationeventsinEastAsiaGenomeBiol.8(11),R245(2007)

edomesticationeventsinEastAsiaGenomeBiol.8(11),R245(2007)
edomesticationeventsinEastAsiaGenomeBiol.8(11),R245(2007)
(12),E28215(2011)
(12),E28215(2011)
(12),E28215(2011)
(12),E28215(2011)
(12),E28215(2011)
(12),E28215(2011)
(12),E28215(2011)
(12),E28215(2011)
(12),E28215(2011)
(12),E28215(2011)
(12),E28215(2011)
(12),E28215(2011)
(12),E28215(2011)
(12),E28215(2011)
(12),E28215(2011)
(12),E28215(2011)
(12),E28215(2011)
(12),E28215(2011)
(12),E28215(2011)
(12),E28215(2011)
(12),E28215(2011)
(12),E28215(2011)
(12),E28215(2011)
(12),E28215(2011)
(12),E28215(2011)
(12),E28215(2011)
(12),E28215(2011)
(12),E28215(2011)
(12),E28215(2011)
(12),E28215(2011)
(12),E28215(2011)
(12),E28215(2011)
(12),E28215(2011)
(12),E28215(2011)
(12),E28215(2011)
(12),E28215(2011)
(12),E28215(2011)
(12),E28215(2011)
(12),E28215(2011)
(12),E28215(2011)
(12),E28215(2011)
(12),E28215(2011)
(12),E28215(2011)
(12),E28215(2011)
(12),E28215(2011)
(12),E28215(2011)
(12),E28215(2011)
tzeriverPLoSONE7(12),E51649(2012)
tzeriverPLoSONE7(12),E51649(2012)
tzeriverPLoSONE7(12),E51649(2012)
tzeriverPLoSONE7(12),E51649(2012)
tzeriverPLoSONE7(12),E51649(2012)
tzeriverPLoSONE7(12),E51649(2012)
tzeriverPLoSONE7(12),E51649(2012)
tzeriverPLoSONE7(12),E51649(2012)
tzeriverPLoSONE7(12),E51649(2012)

tzeriverPLoSONE7(12),E51649(2012)
tzeriverPLoSONE7(12),E51649(2012)
tzeriverPLoSONE7(12),E51649(2012)
tzeriverPLoSONE7(12),E51649(2012)
tzeriverPLoSONE7(12),E51649(2012)
tzeriverPLoSONE7(12),E51649(2012)
tzeriverPLoSONE7(12),E51649(2012)
tzeriverPLoSONE7(12),E51649(2012)
tzeriverPLoSONE7(12),E51649(2012)
tzeriverPLoSONE7(12),E51649(2012)
tzeriverPLoSONE7(12),E51649(2012)
tzeriverPLoSONE7(12),E51649(2012)
tzeriverPLoSONE7(12),E51649(2012)
tzeriverPLoSONE7(12),E51649(2012)
tzeriverPLoSONE7(12),E51649(2012)
tzeriverPLoSONE7(12),E51649(2012)
tzeriverPLoSONE7(12),E51649(2012)
tzeriverPLoSONE7(12),E51649(2012)
tzeriverPLoSONE7(12),E51649(2012)
tzeriverPLoSONE7(12),E51649(2012)
tzeriverPLoSONE7(12),E51649(2012)
tzeriverPLoSONE7(12),E51649(2012)
tzeriverPLoSONE7(12),E51649(2012)
tzeriverPLoSONE7(12),E51649(2012)
tzeriverPLoSONE7(12),E51649(2012)
tzeriverPLoSONE7(12),E51649(2012)
tzeriverPLoSONE7(12),E51649(2012)
tzeriverPLoSONE7(12),E51649(2012)
tzeriverPLoSONE7(12),E51649(2012)
tzeriverPLoSONE7(12),E51649(2012)
tzeriverPLoSONE7(12),E51649(2012)
tzeriverPLoSONE7(12),E51649(2012)
tzeriverPLoSONE7(12),E51649(2012)
tzeriverPLoSONE7(12),E51649(2012)
tzeriverPLoSONE7(12),E51649(2012)
tzeriverPLoSONE7(12),E51649(2012)
tzeriverPLoSONE7(12),E51649(2012)
tzeriverPLoSONE7(12),E51649(2012)
edomesticationeventsinEastAsiaGenomeBiol.8(11),R245(2007)
edomesticationeventsinEastAsiaGenomeBiol.8(11),R245(2007)
edomesticationeventsinEastAsiaGenomeBiol.8(11),R245(2007)
edomesticationeventsinEastAsiaGenomeBiol.8(11),R245(2007)

uencepolymorphismJOURNALAnim.Genet.33(1),19-25(2002)
urredpriortodomesticationJOURNALProc.Biol.Sci.273(1595),1803-1810(2006)
urredpriortodomesticationJOURNALProc.Biol.Sci.273(1595),1803-1810(2006)
urredpriortodomesticationJOURNALProc.Biol.Sci.273(1595),1803-1810(2006)
urredpriortodomesticationJOURNALProc.Biol.Sci.273(1595),1803-1810(2006)
urredpriortodomesticationJOURNALProc.Biol.Sci.273(1595),1803-1810(2006)
tzeriverPLoSONE7(12),E51649(2012)
tzeriverPLoSONE7(12),E51649(2012)
tzeriverPLoSONE7(12),E51649(2012)
tzeriverPLoSONE7(12),E51649(2012)
tzeriverPLoSONE7(12),E51649(2012)
tzeriverPLoSONE7(12),E51649(2012)
tzeriverPLoSONE7(12),E51649(2012)
tzeriverPLoSONE7(12),E51649(2012)
tzeriverPLoSONE7(12),E51649(2012)

tzeriverPLoSONE7(12),E51649(2012)
tzeriverPLoSONE7(12),E51649(2012)
tzeriverPLoSONE7(12),E51649(2012)
tzeriverPLoSONE7(12),E51649(2012)
tzeriverPLoSONE7(12),E51649(2012)
tzeriverPLoSONE7(12),E51649(2012)
tzeriverPLoSONE7(12),E51649(2012)
tzeriverPLoSONE7(12),E51649(2012)
tzeriverPLoSONE7(12),E51649(2012)
tzeriverPLoSONE7(12),E51649(2012)
tzeriverPLoSONE7(12),E51649(2012)
tzeriverPLoSONE7(12),E51649(2012)
tzeriverPLoSONE7(12),E51649(2012)
tzeriverPLoSONE7(12),E51649(2012)
tzeriverPLoSONE7(12),E51649(2012)
tzeriverPLoSONE7(12),E51649(2012)
tzeriverPLoSONE7(12),E51649(2012)
tzeriverPLoSONE7(12),E51649(2012)
tzeriverPLoSONE7(12),E51649(2012)
tzeriverPLoSONE7(12),E51649(2012)
tzeriverPLoSONE7(12),E51649(2012)
tzeriverPLoSONE7(12),E51649(2012)
tzeriverPLoSONE7(12),E51649(2012)
tzeriverPLoSONE7(12),E51649(2012)
tzeriverPLoSONE7(12),E51649(2012)
tzeriverPLoSONE7(12),E51649(2012)
tzeriverPLoSONE7(12),E51649(2012)
tzeriverPLoSONE7(12),E51649(2012)
tzeriverPLoSONE7(12),E51649(2012)
tzeriverPLoSONE7(12),E51649(2012)
tzeriverPLoSONE7(12),E51649(2012)
tzeriverPLoSONE7(12),E51649(2012)
tzeriverPLoSONE7(12),E51649(2012)
tzeriverPLoSONE7(12),E51649(2012)
tzeriverPLoSONE7(12),E51649(2012)
tzeriverPLoSONE7(12),E51649(2012)
tzeriverPLoSONE7(12),E51649(2012)
tzeriverPLoSONE7(12),E51649(2012)
tzeriverPLoSONE7(12),E51649(2012)
tzeriverPLoSONE7(12),E51649(2012)
edomesticationeventsinEastAsiaGenomeBiol.8(11),R245(2007)
edomesticationeventsinEastAsiaGenomeBiol.8(11),R245(2007)
edomesticationeventsinEastAsiaGenomeBiol.8(11),R245(2007)
edomesticationeventsinEastAsiaGenomeBiol.8(11),R245(2007)
edomesticationeventsinEastAsiaGenomeBiol.8(11),R245(2007)
edomesticationeventsinEastAsiaGenomeBiol.8(11),R245(2007)
edomesticationeventsinEastAsiaGenomeBiol.8(11),R245(2007)
edomesticationeventsinEastAsiaGenomeBiol.8(11),R245(2007)
edomesticationeventsinEastAsiaGenomeBiol.8(11),R245(2007)
edomesticationeventsinEastAsiaGenomeBiol.8(11),R245(2007)
edomesticationeventsinEastAsiaGenomeBiol.8(11),R245(2007)
edomesticationeventsinEastAsiaGenomeBiol.8(11),R245(2007)
edomesticationeventsinEastAsiaGenomeBiol.8(11),R245(2007)
edomesticationeventsinEastAsiaGenomeBiol.8(11),R245(2007)
edomesticationeventsinEastAsiaGenomeBiol.8(11),R245(2007)
esGenomics33(2),111-117(2011)
tzeriverPLoSONE7(12),E51649(2012)
tzeriverPLoSONE7(12),E51649(2012)

tzeriverPLoSONE7(12),E51649(2012)
tzeriverPLoSONE7(12),E51649(2012)
tzeriverPLoSONE7(12),E51649(2012)
tzeriverPLoSONE7(12),E51649(2012)
tzeriverPLoSONE7(12),E51649(2012)
tzeriverPLoSONE7(12),E51649(2012)
tzeriverPLoSONE7(12),E51649(2012)
tzeriverPLoSONE7(12),E51649(2012)
tzeriverPLoSONE7(12),E51649(2012)
tzeriverPLoSONE7(12),E51649(2012)
tzeriverPLoSONE7(12),E51649(2012)
tzeriverPLoSONE7(12),E51649(2012)
tzeriverPLoSONE7(12),E51649(2012)
tzeriverPLoSONE7(12),E51649(2012)
tzeriverPLoSONE7(12),E51649(2012)
tzeriverPLoSONE7(12),E51649(2012)
tzeriverPLoSONE7(12),E51649(2012)
tzeriverPLoSONE7(12),E51649(2012)
tzeriverPLoSONE7(12),E51649(2012)
tzeriverPLoSONE7(12),E51649(2012)
tzeriverPLoSONE7(12),E51649(2012)
tzeriverPLoSONE7(12),E51649(2012)
tzeriverPLoSONE7(12),E51649(2012)
tzeriverPLoSONE7(12),E51649(2012)
tzeriverPLoSONE7(12),E51649(2012)
tzeriverPLoSONE7(12),E51649(2012)
tzeriverPLoSONE7(12),E51649(2012)
tzeriverPLoSONE7(12),E51649(2012)
tzeriverPLoSONE7(12),E51649(2012)
tzeriverPLoSONE7(12),E51649(2012)
tzeriverPLoSONE7(12),E51649(2012)
tzeriverPLoSONE7(12),E51649(2012)
tzeriverPLoSONE7(12),E51649(2012)
tzeriverPLoSONE7(12),E51649(2012)
tzeriverPLoSONE7(12),E51649(2012)
tzeriverPLoSONE7(12),E51649(2012)
tzeriverPLoSONE7(12),E51649(2012)
tzeriverPLoSONE7(12),E51649(2012)
tzeriverPLoSONE7(12),E51649(2012)
tzeriverPLoSONE7(12),E51649(2012)
tzeriverPLoSONE7(12),E51649(2012)
tzeriverPLoSONE7(12),E51649(2012)
tzeriverPLoSONE7(12),E51649(2012)
tzeriverPLoSONE7(12),E51649(2012)
tzeriverPLoSONE7(12),E51649(2012)
tzeriverPLoSONE7(12),E51649(2012)
tzeriverPLoSONE7(12),E51649(2012)
edomesticationeventsinEastAsiaGenomeBiol.8(11),R245(2007)
edomesticationeventsinEastAsiaGenomeBiol.8(11),R245(2007)
edomesticationeventsinEastAsiaGenomeBiol.8(11),R245(2007)
edomesticationeventsinEastAsiaGenomeBiol.8(11),R245(2007)
edomesticationeventsinEastAsiaGenomeBiol.8(11),R245(2007)
edomesticationeventsinEastAsiaGenomeBiol.8(11),R245(2007)
edomesticationeventsinEastAsiaGenomeBiol.8(11),R245(2007)
edomesticationeventsinEastAsiaGenomeBiol.8(11),R245(2007)
edomesticationeventsinEastAsiaGenomeBiol.8(11),R245(2007)
edomesticationeventsinEastAsiaGenomeBiol.8(11),R245(2007)
esGenomics33(2),111-117(2011)

edomesticationeventsinEastAsiaGenomeBiol.8(11),R245(2007)
edomesticationeventsinEastAsiaGenomeBiol.8(11),R245(2007)
edomesticationeventsinEastAsiaGenomeBiol.8(11),R245(2007)
edomesticationeventsinEastAsiaGenomeBiol.8(11),R245(2007)
edomesticationeventsinEastAsiaGenomeBiol.8(11),R245(2007)
edomesticationeventsinEastAsiaGenomeBiol.8(11),R245(2007)
tzeriverPLoSONE7(12),E51649(2012)
tzeriverPLoSONE7(12),E51649(2012)
tzeriverPLoSONE7(12),E51649(2012)
tzeriverPLoSONE7(12),E51649(2012)
tzeriverPLoSONE7(12),E51649(2012)
tzeriverPLoSONE7(12),E51649(2012)
tzeriverPLoSONE7(12),E51649(2012)
tzeriverPLoSONE7(12),E51649(2012)
tzeriverPLoSONE7(12),E51649(2012)
tzeriverPLoSONE7(12),E51649(2012)
tzeriverPLoSONE7(12),E51649(2012)
tzeriverPLoSONE7(12),E51649(2012)
tzeriverPLoSONE7(12),E51649(2012)
tzeriverPLoSONE7(12),E51649(2012)
tzeriverPLoSONE7(12),E51649(2012)
tzeriverPLoSONE7(12),E51649(2012)
tzeriverPLoSONE7(12),E51649(2012)
tzeriverPLoSONE7(12),E51649(2012)
tzeriverPLoSONE7(12),E51649(2012)
tzeriverPLoSONE7(12),E51649(2012)
tzeriverPLoSONE7(12),E51649(2012)
tzeriverPLoSONE7(12),E51649(2012)
tzeriverPLoSONE7(12),E51649(2012)
tzeriverPLoSONE7(12),E51649(2012)
tzeriverPLoSONE7(12),E51649(2012)
tzeriverPLoSONE7(12),E51649(2012)
tzeriverPLoSONE7(12),E51649(2012)
tzeriverPLoSONE7(12),E51649(2012)
tzeriverPLoSONE7(12),E51649(2012)
tzeriverPLoSONE7(12),E51649(2012)
tzeriverPLoSONE7(12),E51649(2012)
tzeriverPLoSONE7(12),E51649(2012)
tzeriverPLoSONE7(12),E51649(2012)
tzeriverPLoSONE7(12),E51649(2012)
tzeriverPLoSONE7(12),E51649(2012)
tzeriverPLoSONE7(12),E51649(2012)
tzeriverPLoSONE7(12),E51649(2012)
tzeriverPLoSONE7(12),E51649(2012)
tzeriverPLoSONE7(12),E51649(2012)
